# Supplementary material for: De novo designed 3-helix bundle peptides and proteins with controlled topology and stability
Source: Chem Sci. 2025 Aug 27;16(40):18632–41. doi: 10.1039/d5sc05576h (PMC12441935; doi:10.1039/d5sc05576h)
Supplement: SC-016-D5SC05576H-s001 [file SC-016-D5SC05576H-s001.pdf]

Supplementary Information (SI) for Chemical Science.
This journal is © The Royal Society of Chemistry 2025

De novo designed 3-helix bundle peptides and proteins with controlled topology and stability

Xiyue Leng[1], Katherine I. Albanese[1,2,3], Lia R. Golub[1], Arthur A. Norman[4], Jonathan Clayden[1], and Derek N. Woolfson[1,2,4]*

Author Addresses

[1]School of Chemistry, University of Bristol, Cantock's Close, Bristol BS8 1TS, UK

[2]Max Plack-Bristol Centre for Minimal Biology, University of Bristol, Cantock's Close, Bristol BS8 1TS, UK

[3]Department of Chemistry, Wake Forest University, Winston-Salem NC, USA

[4]School of Biochemistry, University of Bristol, Medical Sciences Building, University Walk, Bristol BS8 1TD, UK

*To whom correspondence should be address: d.n.woolfson@bristol.ac.uk

Table of Contents

1. **Methods** **3**
1.1 General 3
1.2 Computational tools 4
1.3 Peptide synthesis and purification 6
1.4 Protein expression and purification 8
1.5 Solution-phase biophysical characterization 9
1.6 Structural characterization 12
2. **Supplementary data** **14**
1.7 Supplementary tables 14
1.8 Supplementary figures 44
3. **References** **62**

Methods

1.1 General

All solvents, chemicals, and reagents were purchased from commercial sources and used without further purification. Fluorenylmethoxycarbonyl(Fmoc)-α-L-amino acids, Rink amide MBHA resin for solid-phase peptide synthesis (SPPS) and N,N-dimethylformamide (DMF) were purchased from Sigma-Aldrich and Cambridge Reagents. Coupling reagents Oxyma Pure and diisopropylcarbodiimide (DIC) were purchased from Fluorochem and Sigma-Aldrich, respectively. Morpholine and trifluoroacetic acid (TFA) were purchased from Sigma-Aldrich; pyridine was from Thermo Fisher; triisopropylsilane (TIPS) was from Acros Organics. Luria-Broth (LB), antibiotics, IPTG, and L-rhamnose were purchased from Thermo Fisher. All other chemicals were reagent grade and purchased from Sigma-Aldrich.

All reference made to clockwise (CW) and anticlockwise (ACW) topology / overall assembly handedness is defined as follows: with the N-terminal helix at 12 o'clock and coming out towards the viewer, the chain trace follows either a CW or ACW path.

1.2 Computational tools

CC+ search

Antiparallel coiled-coil trimers and bundles were analysed through the online CC+ database.[1] Three-helix coiled coils (CCs) with an antiparallel orientation, hetero-oligomeric partnering, canonical sequence repeats, and consisting of many chains of more than 11 residues were searched with a sequence redundancy cut-off of ≤ 50%. Based on the database that was updated on 1st April 2023, 169 CCs were assigned from 285 α-helical sequences. 121 and 48 CCs were assigned anticlockwise and clockwise, respectively, upon visual inspection. Amino-acid profiles were compiled for each position of the heptad repeat, *abcdefg*. The profile was normalised using the expected amino-acid frequencies from SWISS-PROT to give propensities for each residue.

AlphaFold2 and Esmond predictions

AlphaFold2-multimer predictions (AF2) were performed using the ColabFold (version 1.5.2) with seed 000, single sequence mode on, and three recycle steps to return the top 5 rank models.[2,3] Antiparallel CC trimeric peptides were tested in order to assess the predicted models for each possible assembly. The same single sequence mode was used to assess the sc-apCC3 models. Predicted local distance difference test (pLDDT) scores per residue, predicted template modelling score (pTM), and the predicted alignment error (pAE) matrices were collected to evaluate the quality and confidence of each prediction. To further ensure consistency in topology, particularly important when designing clockwise (CW) and anticlockwise (ACW) variants of largely similar related sequences, ESMFold was used as another structure prediction method. This served to verify that all models retained the expected fold and did not diverge significantly from AF2 predictions in overall topology.

MASTER

Method of Accelerated Search for Tertiary Ensemble Representatives (MASTER)[4,5] was used to build fragments (loops) between adjacent helices in the antiparallel trimeric peptides to connect the N-to-C terminus into a single chain protein (Table S5).

The built-in MASTER database was used and the following parameters were set to find matches to the antiparallel queries (the first and last heptad of each chain): rmsdcut=0.5 and wgap='3-10'.

ProteinMPNN

After MASTER, ProteinMPNN[6] (v_48_020; T = 0.2, Cys, Met, Arg, Asn and Gly omitted, 10 sequences, training noise = 0.2 Å, seed 42) was used to optimise the sequences of the loop region. The peptide backbones were fixed throughout the design process. The 10 designs were then run through AF2 and ESMFold. The sequences with the highest pLDDT (> 85) from AF2, and ESMFold models in agreement with the intended topology were selected for experimental characterizations upon visual inspection.

Dictionary of Secondary Structure of Proteins DSSP

Secondary structure standardisation assignments were generated using the DSSP[7,8] algorithm based on AF2 predicted models. DSSP was used to annotate secondary structure elements and assess the extent of predicted helicity across designed sequences. These assignments were used to verify compatibility with the intended three-helix bundle topology.

1.3 Peptide synthesis and purification

Automated microwave Fmoc solid-phase peptide synthesis (SPPS)

Automated microwave SPPS was performed on a Liberty Blue (CEM) synthesiser with inline UV monitoring. The synthesis was performed on a 0.1 mmol scale with side-chain protection of the amino acids as follows: Gln(Trt), Glu(O^{t}Bu), Lys(Boc), Tyr(^{t}Bu), Trp(Boc). Rink amide (MBHA, 0.65 mmol/g loading, 100-200 mesh) was used for a synthesis on a 0.1 mmol scale. The coupling reactions were performed by adding amino acids dissolved in DMF (2.5 mL, 0.2 M), the coupling reagent DIC in DMF (1.0 mL, 1 M) and Oxyma Pure in DMF (1 mL, 0.5 M) to the respective resin. Standard couplings were performed at 90 °C for 4.5 min (100 W for 20 s, 60 W for 10 s, 35 W for 240 s). Standard deprotections were performed using 20% (v/v) morpholine in DMF at 90 °C for 1.5 min (125 W 30 s, 32 W 60 s). All peptides were manually acetyl capped through addition of pyridine (0.5 mL) and acetic anhydride (0.25 mL) in DMF (9.25 mL), shaking at room temperature (rt) for 20 minutes. The resin was washed 3 times with DMF followed by 6 times with DCM before cleavage. Peptides were cleaved from the resin with addition of 10 mL of a mixture 95:2.5:2.5 *v/v* TFA / H_2O / TIPS, shaking at rt for 2 hours. The TFA solution was then filtered to remove the resin beads and was reduced in volume to ≈ 5 mL or lower using a flow of N_2. Cleaved peptide was precipitated with cold diethyl ether (≈ 45 mL), isolated *via* centrifugation and dissolved in a 1:1 mixture MeCN/H_2O. Crude peptides were lyophilised to yield a white or off-white powder. Crude peptides were kept in -20 °C for short-term storage, and -80 °C for long-term storage.

Semi-preparative scale high-performance liquid chromatography (HPLC)

All peptides were purified by reverse phase HPLC (JASCO) using a Luna C18 (Phenomenex) column (150 x 10 mm^2, 5 μM particle size, 100 Å pore size). Crude peptide was dissolved at 7 mg/mL in 20% *v/v* MeCN in H_2O with 0.1% TFA, injected onto the column and eluted with a 3 mL/min linear gradient (20-100%) of MeCN in H_2O with 0.1% TFA each over 40 min. Elution of the peptide was detected with inline UV monitoring at 220 and 280 nm wavelengths simultaneously. A column oven (50 °C) was employed to improve separation. Pure fractions were identified by analytical

HPLC and matrix-assisted laser desorption/ionisation–time of flight (MALDI-TOF) mass spectrometry, then pooled, and lyophilised.

Analytical HPLC

Analytical HPLC traces were obtained using a Jasco 2000 series HPLC system and a Phenomenex Kinetex C18 (100 x 4.6 mm^2, 5 µm particle size, 100 Å pore size) column. Chromatograms were monitored at 220 and 280 nm wavelengths. The linear gradient was 20–100% *v/v* MeCN in water (each containing 0.1% TFA) over 25 min at a flow rate of 1 mL/min. When required, a column oven (50 °C) was employed to assist peptide elution.

Mass spectrometry

Mass spectra were collected on a Bruker UltraFlex MALDI-TOF mass spectrometer operating in positive-ion reflector mode. Peptides were spotted on a ground steel target plate using α-cyano-4-hydroxycinnamic acid dissolved in 1:1 *v/v* MeCN/H_2O as the matrix. Masses quoted are for the monoisotopic mass as the singly protonated species.

1.4 Protein expression and purification

All genes were transformed then recombinantly expressed in *E. coli* Lemo21-DE3 (NEB) cells. Flasks containing 1 L of LB-kanamycin/chloramphenicol and 0.5 mM L-rhamnose were inoculated with 5 mL overnight cultures and incubated to an OD_{600} of ~0.6 at 37 ˚C with 200 rpm shaking. Expression was induced with 0.5 mM IPTG, and cultures were incubated at 37 ˚C overnight with 200 rpm shaking.

Following expression, cultures were pelleted at 4 krpm for 10 minutes. Cell pellets were resuspended in 20 mL lysis buffer (50 mM Tris, pH 7.4, 500 mM NaCl, 30 mM imidazole, 1 mg/mL lysozyme) for 30 min at 37 ˚C. Resuspended pellets were sonicated using a Biologics Model 3000 Ultrasonic homogeniser with settings to 50% power, 90% pulser (1 pulse/second) for 7 minutes, then clarified at 13 krpm for 30 min. The clarified lysate was heat shocked at 65 ˚C for 10 minutes then cooled on ice for 10 minutes before re-clarifying at 13 krpm for 10 minutes.

The expressed proteins were first purified with Ni-affinity chromatography. Filtered lysate was loaded onto an ÄKTAprime plus (GE Healthcare) equipped with a HisTrap-5 mL HP column (Cytiva). His-tagged proteins were eluted at 55% Buffer B (Buffer A: 50 mM Tris, 500 mM NaCl, 30 mM imidazole, pH 7.4; Buffer B: 50 mM Tris, 500 mM NaCl, 300 mM imidazole, pH 7.4) using a step gradient. Fractions were combined and further purified by size exclusion chromatography using a HiLoad 16/600 Superdex 75 pg size exclusion column (GE Healthcare) equilibrated in buffer containing 50 mM sodium phosphate, pH 7.4, 150 mM NaCl. Eluted fractions were pooled, concentrated, and run on SDS-PAGE to confirm identity. Pure fractions were buffer exchanged into crystallisation buffer(20 mM Tris, 50 mM NaCl, pH 8.0).

1.5 Solution-phase biophysical characterisation

Peptide and protein concentration determination

Peptide and protein concentration was determined at 280 nm using a Nanodrop 2000 (ThermoScientific) spectrometer (ε_{280}(Trp) = 5690 cm^{-1}; ε_{275}(Tyr) = 1405 cm^{-1}, Table S3) or an Agilent (Stockport, UK) Cary 100 UV/vis spectrophotometer. For sequences without Trp or Tyr, concentrations were determined using the amide absorbance for each sequence, using values determined by Gruppen and Kuipers[9] using the equation, where $\varepsilon_{\text{peptide}}$ bond is 923 M^{-1}cm^{-1} , and $\varepsilon_{\text{amino}}$ acid corresponds to the molar extinction coefficient of the free amino acid at 214 nm.:

$$\varepsilon_{\text{peptide}}(M^{-1}\text{cm}^{-1}) = \varepsilon_{\text{peptide bond}} \times n_{\text{peptide bonds}} + \sum_{i=1}^{20} \varepsilon_{\text{amino acid}(i)} \times n_{\text{amino acid}(i)}$$

Circular dichroism (CD) spectroscopy

CD data were collected on a JASCO J-810 or J-815 spectropolarimeter fitted with a Peltier temperature controller in the far-UV region. Samples were made up as 100 µM overall assembly or individual peptide solution in phosphate buffered saline (PBS; 8.2 mM sodium phosphate dibasic, 1.8 mM potassium phosphate monobasic, 137 mM sodium chloride, 2.4 mM potassium chloride), pH 7.4 at 5 °C. For the sc-apCC-3 proteins, CD spectra were acquired at 10 µM protein concentration in PBS at 5 °C. Data were collected in a 1 mm quartz cuvette between 190 and 260 nm and the instrument was set as follows: band width 1 nm, data pitch 1 nm, scanning speed 100 nm/min, 1 s response time. Every CD curve was obtained by averaging 8 scans and subtracting the background signal of buffer and cuvette.

For thermal denaturation experiments, peptide unfolding and refolding were monitored at a wavelength of 222 nm. A temperature range of 5–95 °C and a temperature ramp rate of 60 °C per hour were used with the same settings and peptide or protein concentration as above. Chemical denaturation experiments for sc-apCC3-CW-3NTT were performed across a series of fixed guanidinium chloride (GdmHCl) concentrations prepared in PBS. The sc-apCC3-CW-3NTT samples were subjected to temperature ramping in 2 °C steps (heating rate of 2 °C/min) and the CD signal at 222 nm was monitored. The resulting unfolding curves were analysed by fitting to a

two-state model (folded ↔ unfolded), allowing the signal to vary linearly with denaturant concentration ([GdmHCl]).

The spectra were converted from ellipticities (mdeg) to mean residue ellipticities (MRE, deg·cm^2·dmol^{-1}·res^{-1}) by normalising for concentration of peptide bonds and the cell path length using the equation:

$$MRE = \frac{\theta \times 10^6}{c \times l \times n}$$

where the variable θ is the measured difference in absorbed circularly polarised light in millidegrees, c is the micromolar concentration of the compound, l is the path length of the cuvette in mm, and n is the number of amide bonds in the polypeptide.

Analytical ultracentrifugation (AUC)

Analytical ultracentrifugation (AUC) was performed on a Beckman Optima X-LI analytical ultracentrifuge with an An-60-Ti rotor (Beckman-Coulter). Buffer densities, viscosities and peptide partial specific volumes ($\overline{v}$) were calculated using SEDNTERP (http://rasmb.org/sednterp/).

For sedimentation velocity (SV), peptide sample solutions of 310 or 410 μL were prepared in PBS at sample concentration (250 μM for assembly or individual peptide, 50 μM for protein) and placed in a sedimentation velocity cell with an epon or aluminium, 2-channel centrepiece and sapphire windows. The reference channel was loaded with 320 μL (or 420 μL, respectively) of PBS buffer. The samples were centrifuged at 60 krpm at 20 °C, with absorbance scans taken across a radial range of 5.8–7.3 cm at 5 min intervals to a total of 120 scans.

Data from a single run were fitted to a continuous c(s) distribution model using SEDFIT, at 95% confidence level. Residuals for sedimentation velocity experiments are shown as a bitmap in which the greyscale shade indicates the difference between the fit and raw data (residuals < -0.05 black, > 0.05 white). Good fits are uniformly grey without major dark or light streaks.

Sedimentation equilibrium (SE) experiments were performed in triplicate in a six-channel centrepiece (110 μL per channel) channels at 20 °C. The samples were centrifuged at speeds in the range of 44-60 krpm and scans at each recorded speed were duplicated after equilibration for 8 hours. Data were fitted using SEDPHAT to a

single species model. Monte Carlo analysis was performed to give 95% confidence limits.

Fluorescence quenching experiments

Fluorescence quenching experiments were performed following the previously published protocols.[10] Peptide complexes were prepared by sequential mixing of stoichiometric 1:1:1 molar ratios, following the order: acidic (A), basic (B), and neutral (N) peptide analogues solutions at 33 μM in phosphate buffer (8.2 mM potassium phosphate dibasic, 1.8 mM potassium phosphate monobasic), pH 7.4. Chloride ions are known to quench the signal of 4-cyanophenylalanine.[10] This stepwise addition ensured controlled assembly of the heterotrimeric complexes while minimising off-target mixture formation. Samples were prepared at room temperature, annealed at 95 °C for 60 seconds and allowed to cool back to room temperature over 2 h.
Fluorescence experiments were carried out using a JASCO FP-6500 spectrofluorometer with 26.60-F/Q/10quartz cuvette by Starna Scientific. Samples were excited at 240 nm with a bandwidth of 3 nm. Spectra were recorded between 260 and 400 nm with a bandwidth of 1 nm, reading 200 nm/min. A 1 s response time was used with a 0.5 nm data pitch. A manual voltage of 500 V was used to amplify the signal.

1.6 Structural characterisation

Crystal growth

Diffraction-quality peptide and protein crystals were grown using a sitting-drop vapor-diffusion method. Mixtures of acidic, basic and neutral peptides and sc-apCC3 proteins were annealed beforehand. Purified protein in 20 mM Tris pH 8.0, 50 mM NaCl was concentrated to approximately 20 mg/mL. Protein samples were annealed to 90 °C in a heat block (Grant), incubated at 90 °C for 10 min and slowly cooled down to room temperature over 90 min.

Commercially available sparse matrix screens were used (Morpheus®, JCSG-plus™, Structure Screen 1 and 2, Pact Premier™, ProPlex™; Molecular Dimensions), and the drops were dispensed using a robot (Oryx8; Douglas Instruments). For each well of an MRC 2-drop plate, 0.3 µL of peptide or protein solution and 0.3 µL of reservoir solution in parallel with 0.4 µL of the peptide or protein solution and 0.2 µL of reservoir solution were mixed and the plate was incubated at 20 °C. Crystals generally formed within a month, and after looping were soaked in reservoir solution containing 25% glycerol as a cryoprotectant.

Crystals of sc-apCC3 and its variants were obtained by optimisation by seeding. For seeding experiments, 16-20 mg/mL of protein solution were used and for each well of an MRC 2 drop plate, 0.3 µL protein solution, 0.1 µL of seed, and 0.2 µL of reservoir solution in parallel with 0.4 µL of the peptide or protein solution, 0.1 µL of seed, and 0.1 µL of reservoir solution were mixed and the plate was incubated at 20 °C.

Final crystallisation conditions for proteins are provided in Table S7.

X-ray crystal structure determination

Diffraction data for the crystals were obtained at the Diamond Light Source on beamlines I03, I04, and I24.

Data were processed using the automated pipelines: Xia2 pipelines, which ports data through DIALS or MOSFLM to POINTLESS and AIMLESS as implemented in the CCP4 suite[11], or XDS to XSCALE. Final structures were obtained after iterative rounds of model building with COOT and refinement with PHENIX Refine[12]. Solvent-

exposed atoms lacking map density were either deleted or left at full occupancy. Data collection and refinement statistics are provided in Table S8.

Supplementary data

1.7 Supplementary tables

Table S1. Raw amino-acid counts for antiparallel three-helix coiled coils found by the CC+ database[1]

-	a	b	c	d	e	f	g	Sum
A	161	110	128	295	84	156	93	1027
C	31	7	10	17	3	16	15	99
D	8	86	71	10	48	86	60	369
E	27	179	204	32	159	192	137	930
F	72	22	22	49	30	26	38	259
G	25	34	34	58	25	54	20	250
H	29	36	31	15	32	28	36	207
I	205	46	55	196	127	49	95	773
K	26	138	136	22	120	143	128	713
L	431	94	91	382	222	104	241	1565
M	34	24	16	31	43	15	35	198
N	39	66	55	46	47	64	57	374
P	2	7	7	10	6	12	1	45
Q	56	109	104	63	144	106	128	710
R	18	93	85	23	129	98	92	538
S	64	67	75	79	56	96	61	498
T	81	43	57	66	43	76	71	437
V	166	71	54	118	86	51	59	605
W	15	15	10	8	9	10	10	77
Y	53	27	29	39	41	21	61	271
Sum	1543	1274	1274	1559	1454	1403	1438	9945

Table S2. Helical regions identified for antiparallel three-helix coiled coils found by the CC+ database

PDB ID	Helix Range (Start-End:Chain)	Sequence (Registry)
1bg1	151-179:A 199-228:A 270-284:A	VRKRVQDLEQKMKVVENLQDDFDFNYKTL(defgabcdefgabcdefgabcdefgabcd) KMQQLEQMLTALDQMRRSIVSELAGLLSAM(gabcdefgabcdefgabcdefgabcdefga) LAESQLQTRQQIKKL(abcdefgabcdefga)
1e12_ba1	139-150:A 62-75:C 111-125:C	AADIGMCVTGLA(defgabcdefga) IWGATLMIPLVSIS(efgabcdefgabcd) TWALSTPMILLALGL(abcdefgabcdefga)
1env_ba1	6-76:A 124-149:A 2-72:C	IEEILSKIYHIENEIARIKKLIGEARQLLSGIVQQQNNLLRAIEAQQHLLQLTVWGIKQLQARILAVERYL(defgabcdefgabcdefgabcdefgabcdefgabcdefgabcdefgabcdefgabcdefgabcd) INNYTSLIHSLIEESQNQQEKNEQEL(defgabcdefgabcdefgabcdefga) IEDKIEEILSKIYHIENEIARIKKLIGEARQLLSGIVQQQNNLLRAIEAQQHLLQLTVWGIKQLQARILAV(abcdefgabcdefgabcdefgabcdefgabcdefgabcdefgabcdefgabcdefgabcdefga)
1ez3	37-61:A 71-103:A 113-130:A	VEEIRGFIDKIAENVEEVKRKHSAI(abcdefgabcdefgabcdefgabcd) TKEELEELMSDIKKTANKVRSKLKSIEQSIEQE(gabcdefgabcdefgabcdefgabcdefgabcd) LRIRKTQHSTLSRKFVEV(abcdefgabcdefgabcd)
1fio	40-64:A 76-101:A 115-129:A	ISQINRDLDKYDHTINQVDSLHKRL(abcdefgabcdefgabcdefgabcd) LRHSLDNFVAQATDLQFKLKNEIKSA(gabcdefgabcdefgabcdefgabcd) AENSRQRFLKLIQDY(abcdefgabcdefga)
1fs7	355-369:A 379-393:A 417-431:A	AFDNIGKAHLETGKA(defgabcdefgabcd) LKEIRTHIRHAQWRA(abcdefgabcdefga) GNEEAQKARIKLVKV(defgabcdefgabcd)
1gu6	340-357:A 367-384:A 402-419:A	KIKVEDQLVHAHFEAKAA(abcdefgabcdefgabcd) MKPIQDDIRHAQWRWDLA(abcdefgabcdefgabcd) LGTAMDKAADARTKLARL(abcdefgabcdefgabcd)
1gu6	336-357:E 367-384:E 399-419:E	INDLKIKVEDQLVHAHFEAKAA(defgabcdefgabcdefgabcd) MKPIQDDIRHAQWRWDLA(abcdefgabcdefgabcd) LRMLGTAMDKAADARTKLARL(efgabcdefgabcdefgabcd)
1hx1	159-179:B 207-218:B 233-254:B	LKKLKHLEKSVEKIADQLEEL(efgabcdefgabcdefgabcd) VKATIEQFMKIL(abcdefgabcde) SRLKRKGLVKKVQAFLAECDTV(defgabcdefgabcdefgabcd)

1hz4	51-62:A 70-81:A 89-100:A	IVATSVLGEVLH(defgabcdefga)<br>SLALMQQTEQMA(defgabcdefga)<br>YALWSLIQQSEI(defgabcdefga)
1o5h	25-40:B 66-80:B 108-119:B	GAVGSVVGAXACALAE(defgabcdefgabcde)<br>XEEARLKLFDLAKKD(abcdefgabcdefga)<br>VPXDVIRVXKDL(defgabcdefga)
1ocr	19-30:C 42-56:C 31-42:J	TGALSALLMTSG(defgabcdefga)<br>LLMIGLTTNMLTMYQ(defgabcdefgabcd)<br>LYRVTMTLCLGG(gabcdefgabcd)
1qbz	33-75:A 119-144:A 32-71:C	LAGIVQQQQQLLDVVKRQQELLRLTVWGTKNLQTRVTAIEKYL(defgabcdefgabcdefgabcdefgabcdefgabcdefgabcd)<br>VDFLEENITALLEEAQIQQEKNMYEL(defgabcdefgabcdefgabcdefga)<br>LLAGIVQQQQQLLDVVKRQQELLRLTVWGTKNLQTRVTAI(defgabcdefgabcdefgabcdefgabcdefgabcdefga)
1r8i	41-59:A 150-167:A 176-190:A	LEQMAQQLEQLKSQLETQK(abcdefgabcdefgabcde)<br>YNNQMQELSDMQALTEQI(abcdefgabcdefgabcd)<br>IADLQARIQTSQGAI(abcdefgabcdefga)
1u7l	61-86:A 127-155:A 281-317:A	LIVESEELSKVDNQIGASIGKIIEIL(abcdefgabcdefgabcdefgabcde)<br>IKDLITLISNESSQLDADVRATYANYNSA(defgabcdefgabcdefgabcdefgabcd)<br>EQSLRVQLVRLAKTAYVDVFINWFHIKALRVYVESVL(defgabcdefgabcdefgabcdefgabcdefgabcde)
1uur	251-265:A 288-313:A 326-351:A	IYKLLSEQEQTLVQM(defgabcdefgabcd)<br>LKSLSQKQITLSGQMNTEMSALDATK(abcdefgabcdefgabcdefgabcde)<br>LFALKQDLQIQFKQLSLLHNEIQSIL(abcdefgabcdefgabcdefgabcde)
1wp7	147-171:A 459-481:A 144-165:C	LKSSIESTNEAVVKLQETAEKTVYV(abcdefgabcdefgabcdefgabcd)<br>QISSMNQSLQQSKDYIKEAQRLL(gabcdefgabcdefgabcdefga)<br>INKLKSSIESTNEAVVKLQETA(defgabcdefgabcdefgabcd)
1y1u	148-172:A 218-243:A 282-296:A	FEELRLITQDTENELKKLQQTQEYF(abcdefgabcdefgabcdefgabcd)<br>EAQTLQQYRVELAEKHQKTLQLLRKQ(gabcdefgabcdefgabcdefgabcd)<br>LAEIIWQNRQQIRRA(abcdefgabcdefga)
1ztm	147-171:A 151-172:B 460-481:C	LKEAIRDTNKAVQSVQSSIGNLIVA(abcdefgabcdefgabcdefgabcd)<br>IRDTNKAVQSVQSSIGNLIVAI(defgabcdefgabcdefgabcd)<br>LNKAKSDLEESKEWIRRSNQKL(abcdefgabcdefgabcdefga)

2bug	62-73:A 78-91:A 98-109:A	AIYYGNRSLAYL(defgabcdefga) YGYALNDATRAIEL(abcdefgabcdefg) GYYRRAASNMAL(abcdefgabcde)
2c0m	519-530:F 535-548:F 566-577:F	IRSRYNLGISCI(defgabcdefga) HREAVEHFLEALNM(abcdefgabcdefg) IWSTLRLALSML(abcdefgabcde)
2cwo	13-24:A 39-50:A 78-89:A	ALSRSESLLRRV(defgabcdefga) CVDEFNELASFN(defgabcdefga) IGEMLKEIRAFL(defgabcdefga)
2d4c	29-43:D 151-165:D 197-211:D	LDDDFKEMERKVDVT(defgabcdefgabcd) LREIQSALQHHLKKL(abcdefgabcdefga) SKEIAESSMFNLLEM(defgabcdefgabcd)
2e2a	10-30:C 39-50:C 76-93:C	GFEIVAYAGDARSKLLEALKA(abcdefgabcdefgabcdefg) ADSLVVEAGSCI(defgabcdefga) MMHGQLHLMTTILLKDVI(abcdefgabcdefgabcd)
2ein	19-34:C 42-56:C 31-42:J	TGALSALLMTSGLTMW(defgabcdefgabcde) LLMIGLTTNMLTMYQ(defgabcdefgabcd) LYRVTMTLCLGG(gabcdefgabcd)
2fyz	130-172:A 126-168:C 452-474:D	AQTNARAIAAMKNSIQATNRAVFEVKEGTQRLAIAVQAIQDHI (defgabcdefgabcdefgabcdefgabcdefgabcdefgabcd) SLVQAQTNARAIAAMKNSIQATNRAVFEVKEGTQRLAIAVQAI (abcdefgabcdefgabcdefgabcdefgabcdefgabcdefga) ELSKVNASLQNTVKYIKESNHQL(gabcdefgabcdefgabcdefga)
2gnx	193-204:A 212-223:A 258-269:A	VLSHLLKAQAQI(defgabcdefga) SLVTLHNAHTKL(defgabcdefga) LXKLKTXLLAKF(defgabcdefga)
2hr2	10-21:C 29-40:C 54-65:C	AYLALSDAQRQL(defgabcdefga) AAANCRRAXEIS(defgabcdefga) FDAFCHAGLAEA(defgabcdefga)
2ieq	7-45:A 77-102:A 6-48:C	VASFSSVNDAITQTAEAIHTVTIALNKIQDVVNQQGSAL(abcdefgabcdefgabcdefgabcdefgabcdefgabcd) ELKQLEAKTASLFQTTVELQGLIDQI(gabcdefgabcdefgabcdefgabcd) IVASFSSVNDAITQTAEAIHTVTIALNKIQDVVNQQGSALNHL (abcdefgabcdefgabcdefgabcdefgabcdefgabcdefga)
2iub	175-198:A 207-236:A 251-275:A	DALVDDYFVLLEKIDDEIDVLEEE(efgabcdefgabcdefgabcdefg) TVQRTHQLKRNLVELRKTIWPLREVLSSLY(defgabcdefgabcdefgabcdefgabcde) FRDVYDHTIQIADTVETFRDIVSGL(abcdefgabcdefgabcdefgabcd)

2j7a	372-386:K<br>407-422:K<br>440-460:K	TFDLLLAAQEVSVKA(defgabcdefgabcd)<br>LMIQAREMVRKGQFFW(gabcdefgabcdefga)<br>LDTLAQSQQFSQKAIDLAMEA(efgabcdefgabcdefgabcd)
2n8i	28-39:A 44-58:A 67-81:A	GRALYNIGLEKN(defgabcdefga)<br>VKEAIEYFLRAKKVF(abcdefgabcdefga)<br>ARRAAKSLSEAYQKV(efgabcdefgabcde)
2ntx	113-124:A<br>179-200:A<br>330-352:A	NIPALRKLDAXL(defgabcdefga)<br>LYFQKDSVTQVQKAAXAINAQV(abcdefgabcdefgabcdefga)<br>LESYSRILESLAYTVXSRIEDVL(defgabcdefgabcdefgabcde)
2odu	424-449:A<br>462-486:A<br>501-519:A	IVTKLQMEAGLCEEQLNQADALLQSD(gabcdefgabcdefgabcdefgabcd)<br>AGEVERDLDKADSMIRLLFNDVQTL(abcdefgabcdefgabcdefgabcd)<br>VYRLHKRLVAIRTEYNLRL(abcdefgabcdefgabcde)
2oex	430-457:A<br>492-516:A<br>663-691:A	IQTVDQLIKELPELLQRNREILDESLRL(abcdefgabcdefgabcdefgabcdefg)<br>GTNFRTVLDKAVQADGQVKECYQSH(abcdefgabcdefgabcdefgabcd)<br>FVELVANLKEGTKFYNELTEILVRFQNKC(abcdefgabcdefgabcdefgabcdefga)
2p22	254-286:A<br>89-126:C<br>43-61:D	MQESIARFHEIIAIDKNHLRAVEQAIEQTMHSL(defgabcdefgabcdefgabcdefgabcdefga)<br>AGKIHAFRDQFKQLEENFEDLHEQKDKVQALLENARIL(efgabcdefgabcdefgabcdefgabcdefgabcdefg)<br>TRDLLCPWYEECDNITKVC(defgabcdefgabcdefga)
2qfc	155-166:A<br>174-185:A<br>194-205:A	LYIENAIANIYA(defgabcdefga)<br>GIDLFEQILKQL(defgabcdefga)<br>FDVKVRYNHAKA(defgabcdefga)
3a8y	372-383:D<br>398-409:D<br>427-438:D	VLGNLSEIQGEV(defgabcdefga)<br>LEELLTKQLLAL(defgabcdefga)<br>AVRLAQNILSYL(defgabcdefga)
3atp	56-70:A 90-104:A 129-140:A	SWVALLQTRNTLNRA(defgabcdefgabcd)<br>LMESASISLKQAEKN(gabcdefgabcdefg)<br>YDIYHNALAELI(defgabcdefga)
3c18	122-140:B<br>148-165:B<br>204-215:B	XSLSFAKLLRRFQDGRNLF(defgabcdefgabcdefga)<br>AYTHVHHALHHLARLSVL(defgabcdefgabcdefg)<br>IHLALIGLEHLL(defgabcdefga)
3efz	142-156:B<br>167-182:B<br>194-205:B	AFCIKLKGDLMRYKA(defgabcdefgabcd)<br>CIKQAVEFYEDALQRE(gabcdefgabcdefga)<br>LYLATILNYTIL(defgabcdefga)
3etv	73-84:A 92-103:A 120-131:A	FLNLIKEVKTNL(defgabcdefga)<br>CYYSLQSLRKKM(defgabcdefga)<br>ISTYVDTLHLEL(defgabcdefga)

3f4y	4-35:A 3-31:B 10-31:E	VQQQNNLLRAIEAQQHLLQLTVWGIKQLQARI(abcdefgabcdefgabcdefgabcdefgabcd) IVQQQNNLLRAIEAQQHLLQLTVWGIKQL(abcdefgabcdefgabcdefgabcdefga) IAEYAARIEALIRAAQEQQEKN(defgabcdefgabcdefgabcd)
3fp2	161-172:A 177-190:A 197-208:A	PVFYSNISACYI(defgabcdefga) LEKVIEFTTKALEI(abcdefgabcdefg) ALLRRASANESL(abcdefgabcde)
3h90	80-94:A 145-156:A 190-205:A	SLAALAQSMFISGSA(abcdefgabcdefga) QAVRADMLHYQS(defgabcdefga) ILYSALRMGYEAVQSL(defgabcdefgabcde)
3k1s	8-29:H 38-59:H 74-85:H	IPFQLILNSGNARSFAXEALQF(gabcdefgabcdefgabcdefg) ADEAXVKAKEAINEAHHFQTEL(defgabcdefgabcdefgabcd) LLIHAQDHLXNA(gabcdefgabcd)
3l8r	17-31:C 39-53:C 83-97:C	SGNARSIVHEAFDAM(abcdefgabcdefga) AEQKLQEANDELLKA(defgabcdefgabcd) LMTTMTLREVAIEML(abcdefgabcdefga)
3m71_ba1	15-27:B 41-52:B 85-96:B	GIPLGLAALSLAW(bcdefgabcdefg) SDVLGIVASAVW(defgabcdefga) LIPITTMLVGDI(gabcdefgabcd)
3mx3	113-124:A 160-171:A 190-201:A	GLAHFIKAYKIF(defgabcdefga) ILSDLETAYELF(defgabcdefga) WKKATTAFQLKV(defgabcdefga)
3n27	8-29:A 58-76:A 4-31:C	INKLKSSIESTNEAVVKLQETA(defgabcdefgabcdefgabcd) AKSDLEESKEWIRRSNQKL(defgabcdefgabcdefga) NADNINKLKSSIESTNEAVVKLQETAEK(abcdefgabcdefgabcdefgabcdefg)
3nqp	142-156:A 191-205:A 224-237:A	GEVYGLRAFYYFDLY(defgabcdefgabcd) XTQIKSDLNKSXEYF(abcdefgabcdefga) KAATECLXGEVYLW(efgabcdefgabcd)
3okq_ba1	562-587:A 603-670:A 603-673:B	SQTELGDLSDTLLSKVDDLQDVIEIM(gabcdefgabcdefgabcdefgabcd) LETVSKDLENAQADVLKLQEFIDTEKPHWKKTWEAELDKVCEEQQFLTLQEELILDLKEDLGKALETF(abcdefgabcdefgabcdefgabcdefgabcdefgabcdefgabcdefgabcdefgabcdefgabcde) LETVSKDLENAQADVLKLQEFIDTEKPHWKKTWEAELDKVCEEQQFLTLQEELILDLKEDLGKALETFDLI(defgabcdefgabcdefgabcdefgabcdefgabcdefgabcdefgabcdefgabcdefgabcdefgabcd)
3q15	139-150:A 158-169:A 178-189:A	AEFHFKVAEAYY(defgabcdefga) SMYHILQALDIY(defgabcdefga) RTIQSLFVIAGN(defgabcdefga)

3sxq	434-455:A 468-479:A 497-518:A	THGKIMKAEFWLARMIDLFPVA(defgabcdefgabcdefgabcd) VRALHYDAHLHW(defgabcdefga) RESLMKSITKSKEGVGKLDAAI(efgabcdefgabcdefgabcde)
3txn	208-219:A 228-239:A 246-257:A	GALDLQSGILHA(defgabcdefga) AFSYFYEAFEGF(defgabcdefga) KALTSLKYMLLC(defgabcdefga)
3ulq	141-152:A 160-171:A 180-191:A	AEFFFKMSESYY(defgabcdefga) SMDYARQAYEIY(defgabcdefga) RLLQCHSLFATN(defgabcdefga)
3um3	240-261:A 270-291:A 309-323:A	KYLHLKMCFYTAYAYCYHGETL(gabcdefgabcdefgabcdefg) AIRSLQEAEKLYAKAEALCKEY(defgabcdefgabcdefgabcd) FRKLGNLVKNTLEKC(abcdefgabcdefga)
3uun	352-365:A 372-389:A 427-452:A	LSWLLSAEDTLQAQ(efgabcdefgabcd) VEVVKDQFHTHEGYMMDL(efgabcdefgabcdefga) LNSRWECLRVASMEKQSNLHRVLMDL(defgabcdefgabcdefgabcdefga)
3vtq	38-69:E 37-65:B 128-145:D	VQQQNNLLRAIEAQQHLLQLTVWGIKQLQARI(abcdefgabcdefgabcdefgabcdefgabcd) IVQQQNNLLRAIEAQQHLLQLTVWGIKQL(abcdefgabcdefgabcdefgabcdefga) TKQIYKILEESQEQQDRN(abcdefgabcdefgabcd)
3wmi_ba1	487-526:A 572-599:B 502-523:E	VQNHTFEVENNTINGLELVEEQVHILYAMVLQTHADVQLL(defgabcdefgabcdefgabcdefgabcdefgabcdefga) VDKMENLNHDILTTLHTARNNLEQSMIT(abcdefgabcdefgabcdefgabcdefg) LELVEEQVHILYAMVLQTHADV(defgabcdefgabcdefgabcd)
3zc1	23-38:D 46-60:D 77-88:D	VLREMRIHSTKSIALI(gabcdefgabcdefga) AEQELKKAIELLEKV(defgabcdefgabcd) AMQELVEAIAFK(defgabcdefga)
4a1s	157-178:B 190-211:B 217-230:B	RLSEGRALYNLGNVYHAKGKHL(gabcdefgabcdefgabcdefg) VKEALTRAVEFYQENLKLMRDL(defgabcdefgabcdefgabcd) QGRACGNLGNTYYL(efgabcdefgabcd)
4e68	151-179:A 199-228:A 270-287:A	VRKRVQDLEQKMKVVENLQDDFDFNYKTL(defgabcdefgabcdefgabcdefgabcd) KMQQLEQMLTALDQMRRSIVSELAGLLSAM(gabcdefgabcdefgabcdefgabcdefga) LAESQLQTRQQIKKLEEL(abcdefgabcdefgabcd)
4egw	144-161:A 177-198:A 221-241:A	TRSYSRILMNLEDELEEL(abcdefgabcdefgabcd) ILGLRKTLVYFHKSLIANRDVL(abcdefgabcdefgabcdefga) YYDTLQLIDMSATYREVLTSM(efgabcdefgabcdefgabcd)

4eqf	201-212:A<br>217-230:A<br>237-248:A	YSLWNRLGATLA(defgabcdefga)<br>SEEAVEAYTRALEI(abcdefgabcdefg)<br>SRYNLGISCINL(abcdefgabcde)
4ev6	144-161:A<br>174-202:A<br>217-245:A	TRSYSRILMNLEDELEEL(abcdefgabcdefgabcd)<br>MEKILGLRKTLVYFHKSLIANRDVLVLLK(efgabcdefgabcdefgabcdefgabcde)<br>FEDLYYDTLQLIDMSATYREVLTSMMDIT(abcdefgabcdefgabcdefgabcdefga)
4f53	134-145:A<br>179-190:A<br>213-224:A	SLAELYRIVVXL(defgabcdefga)<br>XLEDLDNIITVL(defgabcdefga)<br>WYKFANSLKLRX(defgabcdefga)
4gpk	189-200:A<br>208-219:A<br>227-238:A	TGLYYNIALAYT(defgabcdefga)<br>AIHFVNMALEGF(defgabcdefga)<br>NIINCQILIAVS(defgabcdefga)
4gpk	229-240:I<br>245-259:I<br>268-283:I	INCQILIAVSYT(defgabcdefga)<br>YEEALKMYESILREA(abcdefgabcdefga)<br>LLAITLSNMGSIYYKK(defgabcdefgabcde)
4hwc	164-175:C<br>196-207:C<br>223-234:C	ISLEVDRLGGRV(defgabcdefga)<br>VIELLMNELIKL(defgabcdefga)<br>QVKRVQNYVETL(defgabcdefga)
4hwd	144-158:D<br>170-187:D<br>203-221:D	ISFQVERLAGQLSAF(defgabcdefgabcd)<br>EKNLENLMEMLMNQLVKL(efgabcdefgabcdefga)<br>QEERLHKYVEALDLLKIKN(defgabcdefgabcdefga)
4hwh	146-159:C<br>174-185:C<br>204-218:C	TGEVDKLSDRVVAL(efgabcdefgabcd)<br>FDMAAELLMRQL(abcdefgabcde)<br>EVRRIQNLQEAVDKL(defgabcdefgabcd)
4i0u	167-198:C<br>207-232:C<br>247-275:C	DYLLYSLIDALVDDYFVLLEKIDDEIDVLEEE(defgabcdefgabcdefgabcdefgabcdefg)<br>TVQRTHQLKRNLVELRKTIWPLREVL(defgabcdefgabcdefgabcdefga)<br>TVPYFRDVYDHTIQIADTVETFRDIVSGL(defgabcdefgabcdefgabcdefgabcd)
4jhr	131-149:B<br>171-188:B<br>194-207:B	KVGEARALYNLGNVYHAKG(gabcdefgabcdefgabcd)<br>LQAAVDLYEENLSLVTAL(abcdefgabcdefgabcd)<br>QGRAFGNLGNTHYL(efgabcdefgabcd)
4kik	451-469:A<br>533-544:A<br>612-629:A	QRAAMMNLLRNNSCLSKMK(defgabcdefgabcdefga)<br>LVERMMALQTDI(defgabcdefga)<br>LSKTVVCKQKALELLPKV(efgabcdefgabcdefga)
4nqj	168-257:A<br>273-287:A<br>160-257:C	QGQLETTLKELQTLRNMQKEAIAAHKENKLHLQQHVSMEFLKLHQFLHSKEKDILTELREEGKALNEEMELNLSQLQEQCLLAKDMLVSI(cdefgabcdefgabcdefgabcdefgabcdefgabcdefgabcdefgabcdefgabcdefgabcdefgabcdefgabcdefgabcdefgabcdefga) ITTLLHSLEQGMKVL(defgabcdefgabcd)<br>FMEELAIQQGQLETTLKELQTLRNMQKEAIAAHKENKLHLQQH

		VSMEFLKLHQFLHSKEKDILTELREEGKALNEEMELNLSQLQE QCLLAKDMLVSI(defgabcdefgabcdefgabcdefgabcde fgabcdefgabcdefgabcdefgabcdefgabcdefgabcdef gabcdefgabcdefgabcdefgabc)
4p1n	660-678:A 711-728:A 472-483:C	KKLCMESLLLYLKSLTILA(defgabcdefgabcdefga) FNECLDKAEFLRLKLHTL(defgabcdefgabcdefg) ISSSLIKFQSMK(defgabcdefga)
4r61	16-48:A 64-96:A 127-159:A	LLRQIEAQQHLLQLTVSKIKQLQARILAVERYL(defgabcde fgabcdefgabcdefgabcdefga) LYREVALIRAQLQKIESETLQLLHQQAEIEREL(defgabcde fgabcdefgabcdefgabcdefga) LKRAIEAQKHLLQLTVWGIKQLQARILAVERYL(defgabcde fgabcdefgabcdefgabcdefga)
4u6u_ba3	46-71:A 111-136:B 111-139:H	CSSLLSKMDYYSGHITKELESTIQVL(gabcdefgabcdefga bcdefgabcd) FERALKLQTVSSKIHQTTTLLRSSLI(abcdefgabcdefgab cdefgabcde) FERALKLQTVSSKIHQTTTLLRSSLIYVH(defgabcdefgab cdefgabcdefgabcd)
4ui9	703-714:F 719-732:F 739-750:F	PLCKFHRASVLF(defgabcdefga) YKSALQELEELKQI(abcdefgabcdefg) VYFLIGKVYKKL(abcdefgabcde)
4ui9	581-592:O 600-611:O 619-630:O	ISVLLSVAELYW(defgabcdefga) ALPMLLQALALS(defgabcdefga) LASETVLNLAFA(defgabcdefga)
4ui9	661-675:O 686-701:O 710-724:O	GRAMFLVAKCQVASA(defgabcdefgabcd) ALEAAIENLNEAKNYF(gabcdefgabcdefga) IRDVVYFQARLYHTL(efgabcdefgabcde)
4wpc	45-80:A 113-148:A 237-248:A	INALLSRLKQSLLTCEEFMKFIRKKYAFEEEHVQEL(defgab cdefgabcdefgabcdefgabcdefgabcd) AQVKQSYITALQKMYSEISSLLLTMTKLRKSVKENS(abcdef gabcdefgabcdefgabcdefgabcdefga) IVQELKDLILEI(defgabcdefga)
4xng	89-102:B 114-127:B 181-199:B	LVGLQQLELEYVNL(efgabcdefgabcd) TELLNNLKELVDEH(efgabcdefgabcd) LRQRFKKLSQKIDSSLKQI(defgabcdefgabcdefga)
4ynw	254-265:A 270-283:A 290-301:A	FPSRVSLVRLLI(defgabcdefga) EEEALEVTERLIAE(abcdefgabcdefg) VWYLGGYARYRL(abcdefgabcde)
4yo5	436-447:C 455-466:C 478-489:C	WLLRLLXARVAE(defgabcdefga) ALHLLAELDERA(defgabcdefga) LVFEVKARRLKL(defgabcdefga)
4yv6	185-196:D 204-215:D 223-234:D	TQLSINCLIISI(defgabcdefga) SHYLIEKIEFLL(defgabcdefga) EKTVFLYVHGYY(defgabcdefga)

5a7d	298-309:C 314-328:C 337-351:C	AQSCYSLGNTYT(defgabcdefga) FNTAIEYHNRHLAIA(abcdefgabcdefga) EARACWSLGNAHSAI(efgabcdefgabcde)
5aqf	159-179:B 207-221:B 233-254:B	LKKLKHLEKSVEKIADQLEEL(efgabcdefgabcdefgabcd) VKATIEQFMKILEEI(abcdefgabcdefga) SRLKRKGLVKKVQAFLAECDTV(defgabcdefgabcdefgabcd)
5aqg	159-176:B 207-221:B 233-255:B	LKKLKHLEKSVEKIADQL(efgabcdefgabcdefga) VKATIEQFMKILEEI(abcdefgabcdefga) SRLKRKGLVKKVQAFLAECDTVE(defgabcdefgabcdefgabcde)
5cwi	160-171:A 188-204:A 214-228:A	LASQAAEAVKLA(defgabcdefga) IKAASEAAEEASKAAEE(efgabcdefgabcdefg) ARDEIKEASQKAEEV(defgabcdefgabcd)
5cwm	3-24:A 33-54:A 62-83:A	PEDELKRVEKLVKEAEELLRQA(defgabcdefgabcdefgabcd) LEKALRTAEEAAREAKKVLEQA(abcdefgabcdefgabcdefga) VALRAVELVVRVAELLLRIAKE(defgabcdefgabcdefgabcd)
5cwo	67-81:A 89-100:A 120-134:A	AAKVAAEVIKVAIQA(abcdefgabcdefga) LFRAALELVRAV(defgabcdefga) ARAAKVAAEVIKVAI(abcdefgabcdefga)
5cwq	33-51:A 70-84:A 92-109:A	LKELAEALIEEARAVQELA(defgabcdefgabcdefga) AQRVLEEARKVSEEA(abcdefgabcdefga) VLALALIAIALAVLALAE(defgabcdefgabcdefg)
5ehb_ba1	4-21:A 8-25:B 8-26:C	IXDALEKLAEIQKEIAEF(abcdefgabcdefgabcd) LEKLAEIQKEIAEFLREL(efgabcdefgabcdefga) LEKLAEIQKEIAEFLRELI(defgabcdefgabcdefga)
5gjq	167-178:Q 186-197:Q 206-217:Q	VEVQLLESKTYH(defgabcdefga) ARAALTSARTTA(defgabcdefga) LQATLDMQSGII(defgabcdefga)
5h0n	18-39:A 61-75:B 14-42:E	QNNLLRAIEAQQHLLQLTVWGI(defgabcdefgabcdefgabcd) IEEYTKKIEELIKKS(defgabcdefgabcd) IVQQQNNLLRAIEAQQHLLQLTVWGIKQL(abcdefgabcdefgabcdefgabcdefga)
5h6i	50-61:A 80-91:A 104-115:A	MAQKLDQDSIQL(defgabcdefga) AITSVEKLKTSL(defgabcdefga) IGSRVEALTDVI(defgabcdefga)
5iig	70-81:A 102-113:A 163-174:A	VFRRVKEVQEQV(defgabcdefga) LEEELSDIIADV(defgabcdefga) LVVKISQLYDIA(defgabcdefga)

5ir4	103-121:A 132-150:A 174-192:A	VDWLLKTAGVIVELIVNFV(defgabcdefgabcdefga) FERIAAGLSGDLEAARQVH(defgabcdefgabcdefga) LQTRVIALLTRVGLLVDDI(defgabcdefgabcdefga)
5j0j	5-30:A 42-70:A 46-67:C	IKETLKRLEDSLRELRRILEELKEML(defgabcdefgabcdefgabcdefga) IVEVLKVIVKAIEASVENQRISAENQKAL(abcdefgabcdefgabcdefgabcdefga) LKVIVKAIEASVENQRISAENQ(defgabcdefgabcdefgabcd)
5j1h	758-769:B 787-811:B 896-921:B	VREAEGQLQKLQ(abcdefgabcde) LEDLLQDAQDEKEQLNEYKGHLSGL(abcdefgabcdefgabcdefgabcd) VTRLEAQHQALVTLWHQLHVDMKSLL(abcdefgabcdefgabcdefgabcde)
5j1i	1013-1034:A 1046-1067:A 1090-1119:A	ISELKDIRLQLEACETRTVHRL(efgabcdefgabcdefgabcde) CAQRIAEQQKAQAEVEGLGKGV(abcdefgabcdefgabcdefga) LRSELELTLGKLEQVRSLSAIYLEKLKTIS(defgabcdefgabcdefgabcdefgabcde)
5j2l	10-34:B 41-69:B 48-73:A	QRELDKQRKDTEEIRKRLKEIQRLT(efgabcdefgabcdefgabcdefga) ADELIKELREIIRRLQEQSEKLREIIEEL(abcdefgabcdefgabcdefgabcdefga) LREIIRRLQEQSEKLREIIEELEKII(defgabcdefgabcdefgabcdefga)
5j2l	41-69:B 6-34:A 44-72:A	ADELIKELREIIRRLQEQSEKLREIIEEL(defgabcdefgabcdefgabcdefgabcd) LREAQRELDKQRKDTEEIRKRLKEIQRLT(abcdefgabcdefgabcdefgabcdefga) LIKELREIIRRLQEQSEKLREIIEELEKI(defgabcdefgabcdefgabcdefgabcd)
5l1x	456-481:H 119-165:J 120-162:L	FNVALDQVFESIENSQALVDQSNRIL(defgabcdefgabcdefgabcdefga) TAGVAIAKTIRLESEVTAIKNALKKTNEAVSTLGNGVRVLATAVREL(defgabcdefgabcdefgabcdefgabcdefgabcdefgabcdefga) AGVAIAKTIRLESEVTAIKNALKKTNEAVSTLGNGVRVLATAV(defgabcdefgabcdefgabcdefgabcdefgabcdefgabcd)
5lki	1857-1868:C 1879-1890:C 1959-1970:C	LDLLIARGDHAY(defgabcdefga) AKMWYMQALHLL(defgabcdefga) YWQTLAQRVYNL(defgabcdefga)
5mj3	7-25:C 51-69:C 95-113:C	IQKEIAQIQAVIAGIQKYI(defgabcdefgabcdefga) IQKQIAAIQXQIAAIQKQI(defgabcdefgabcdefga) IQKQIAAIQEQILAIYKQI(defgabcdefgabcdefga)

5mjy	243-254:B 273-286:B 298-309:B	MKIYYFAAVAHL(defgabcdefga) FQSALDKLNEAIKL(abcdefgabcdefg) LRFTMDVIGGKY(abcdefgabcde)
5n77	131-162:A 174-203:A 212-244:A	ELLLDLFETKIEQLADEIENIYSDLEQLSRVI(efgabcdefgabcdefgabcdefgabcdefga) ALSTLAELEDIGWKVRLCLMDTQRALNFLV(defgabcdefgabcdefgabcdefgabcde) QLEQAREILRDIESLLPHNESLFQKVNFLMQAA(defgabcdefgabcdefgabcdefgabcdefga)
5t5s	14-25:A 35-46:A 59-70:A	LKKCLSVMEAKV(defgabcdefga) VQREIADLGEAL(defgabcdefga) LRETLKSLKKVM(defgabcdefga)
5vjx	15-43:U 17-46:V 21-42:W	ITLKTKELIRQNQATQAELDQLKEQTQXF(defgabcdefgabcdefgabcdefgabcd) LKDQLEQRTRXIEANIHRQQEELRKIQEQL(gabcdefgabcdefgabcdefgabcdefga) LEQRTRXIEANIHRQQEELRKI(defgabcdefgabcdefgabcd)
5xdb	249-260:A 309-320:A 337-348:A	PITQLIFTHLYL(defgabcdefga) AEAGAREVALRV(defgabcdefga) LMVQVASAKIVA(defgabcdefga)
5xdb	249-267:C 302-320:C 337-355:C	PITQLIFTHLYLGIARGAL(defgabcdefgabcdefga) FAAQLQVAEAGAREVALRV(defgabcdefgabcdefga) LMVQVASAKIVATRLVIEL(defgabcdefgabcdefga)
5xsj	141-156:L 171-186:L 197-211:L	ITNEIKQHVDSSLDNF(gabcdefgabcdefga) YNNEVILAKQKIGNLK(defgabcdefgabcde) LRDLDNTLDSYIESS(abcdefgabcdefga)
5z2c	294-311:A 325-336:A 350-367:A	LFVLTAVNIRGTCLLSYS(abcdefgabcdefgabcd) LCEAKEAFEIGL(abcdefgabcde) ELHSFVKAAFGLTTVHRR(abcdefgabcdefgabcd)
5z2c	194-205:B 213-224:B 234-245:B	SVCIQIRGQILQ(defgabcdefga) AAELIWASIVGY(defgabcdefga) GLSTSLGILADI(defgabcdefga)
5zhy	10-49:A 108-132:A 6-52:C	FNKAMTNIVDAFTGVNDAITQTSQALQTVATALNKIQDVV(gabcdefgabcdefgabcdefgabcdefgabcdefgabcd) ISTLENKSAELNYTVQKLQTLIDNI(abcdefgabcdefgabcdefgabcd) LAASFNKAMTNIVDAFTGVNDAITQTSQALQTVATALNKIQDVVNQQ(defgabcdefgabcdefgabcdefgabcdefgabcdefgabcdefga)
6c12	459-471:B 483-494:B 516-527:B	IRKALQECMQHNF(defgabcdefgab) GLEQLKVIRERL(defgabcdefga) LDNLMETAYATA(defgabcdefga)

6dkm	45-69:C 87-111:D 121-141:D	ERLLEELLRILDENAELLKRNLELL(abcdefgabcdefgabcdefgabcd)<br>LREYHRVLREYEKLLEELRRLYEEY(abcdefgabcdefgabcdefgabcd)<br>SDRILREIKEILDKSERLWDL(abcdefgabcdefgabcdefg)
6ds9	7-25:A 35-56:A 70-92:A	FKQRLAAIKTRLAAIKTRL(defgabcdefgabcdefga)<br>LAAFEKEIAAFESEIAAFESEL(abcdefgabcdefgabcdefga)<br>LRKEAAAIRDEAAAIRDELQAYR(defgabcdefgabcdefgabcde)
6efk	94-105:B 110-123:B 135-146:B	VKAHFFLGQCQL(defgabcdefga)<br>YDEAIANLQRAYSL(abcdefgabcdefg)<br>IPSALRIAKKKR(abcdefgabcde)
6gy7	298-316:A 119-133:D 302-319:D	LTEAVKIEQVWISFAEQLH(gabcdefgabcdefgabcd)<br>DNVIVSFTGRTSKLT(abcdefgabcdefga)<br>VKIEQVWISFAEQLHKLS(abcdefgabcdefgabcd)
6gyp	344-355:B 373-384:B 403-414:B	LYWKIISLDRDL(defgabcdefga)<br>IRRELDIFQYKV(defgabcdefga)<br>ALFQISTVSWKL(defgabcdefga)
6mbw	151-162:A 229-240:A 285-296:A	LRLVTQDTENEL(defgabcdefga)<br>LAEKHQKTLQLL(defgabcdefga)<br>IIWQNRQQIRRA(defgabcdefga)
6mbz	230-244:B 282-296:B 313-327:B	AEKHQKTLQLLRKQQ(abcdefgabcdefga)<br>LAEIIWQNRQQIRRA(defgabcdefgabcd)<br>LAEVNATITDIISAL(abcdefgabcdefga)
6n9h_ba1	2-31:A 43-74:A 40-65:C	AQDKLKYLVKQLERALRELKKSLDELERSL(gabcdefgabcdefgabcdefgabcdefga)<br>LVENNRLNVENNKIIVEVLRIILELAKASAKL(abcdefgabcdefgabcdefgabcdefgabcd)<br>EDALVENNRLNVENNKIIVEVLRIIL(defgabcdefgabcdefgabcdefga)
6nqx	103-114:C 164-175:C 192-203:C	ALSNYQSGVNTI(defgabcdefga)<br>ALNYFSKAINHL(defgabcdefga)<br>LLSDVYRSWIMA(defgabcdefga)
6nt9	450-471:A 505-526:A 626-647:A	IKDDYNETVHKKTEVVITLDFC(defgabcdefgabcdefgabcd)<br>LLRLSSSQGTIETSLQDIDSRL(abcdefgabcdefgabcdefga)<br>LRKQLLSLTNQCFDIEEEVSKY(defgabcdefgabcdefgabcd)
6nz3	27-86:A 4-21:B 30-89:B	EKQIVEAIRAIVENNAQIVEAIRAIVENNAQIVENNRAIIEALEAIGGHNKILEEMKKQL(defgabcdefgabcdefgabcdefgabcdefgabcdefgabcdefgabcdefgabcdefg)<br>MGDLKYSLERLREILERL(abcdefgabcdefgabcd)<br>IVEAIRAIVENNAQIVEAIRAIVENNAQIVENNRAIIEALEAI

		GGHNKILEEMKKQLKDL(abcdefgabcdefgabcdefgabcdefgabcdefgabcdefgabcdefgabcdefgabcd)
6poo	307-329:A 342-353:A 367-388:A	YAKKIERISSKGLALSKKAKEIY(gabcdefgabcdefgabcdefga) YADSVGTYLNRF(defgabcdefga) GQSGLDEAKKXLDEVKKLLKEL(abcdefgabcdefgabcdefga)
6q6g	445-456:Q 461-474:Q 481-492:Q	EPLLNNLGHVCR(defgabcdefga) YAEALDYHRQALVL(abcdefgabcdefg) TYSAIGYIHSLM(abcdefgabcde)
6qdl	81-92:A 97-110:A 117-128:A	VKALFRRSLARE(defgabcdefga) VGPAFQDAKEALRL(abcdefgabcdefg) IVEVLQRLVKAN(abcdefgabcde)
6qti	657-668:A 709-720:A 788-799:A	PQLVAAFHSLVG(defgabcdefga) TFSGSLVAYGKL(defgabcdefga) MPVVITVLNSYS(defgabcdefga)
6r17	63-143:A 67-143:B 78-103:D	LDSSIDILQKRAQELIENINKSRQKDHALMTNFRNSLKTKVSDLTEKLEERIYQIYNDHNKIIQEKLQEFTQKMAKISHLE(defgabcdefgabcdefgabcdefgabcdefgabcdefgabcdefgabcdefgabcdefgabcdefgabcdefgabcdefg) IDILQKRAQELIENINKSRQKDHALMTNFRNSLKTKVSDLTEKLEERIYQIYNDHNKIIQEKLQEFTQKMAKISHLE(bcdefgabcdefgabcdefgabcdefgabcdefgabcdefgabcdefgabcdefgabcdefgabcdefgabcdefga) RAAVDASYIDEIDELFKEANAIENFL(defgabcdefgabcdefgabcdefga)
6r80	936-951:A 994-1005:A 1145-1159:A	FEKAVYYLDAVVSFIE(defgabcdefgabcde) ADKRLTVLCLRC(defgabcdefga) VRYTRQGLHWLRQDA(abcdefgabcdefga)
6re5	241-252:2 262-273:2 284-295:2	PAQAVEAAYGLA(defgabcdefga) FKALFGVVAPAI(defgabcdefga) SLAQLHVASTIS(defgabcdefga)
6rw6	2019-2043:C 2255-2279:C 2307-2318:C	FPHMLENARGMVSQLTQFGSTLQNI(defgabcdefgabcdefgabcdefg) RGRLAAIYFQFYDLAVARCLMAEQA(defgabcdefgabcdefgabcdefg) GETLMLSLAQME(abcdefgabcde)
6rw9	1988-2005:A 2223-2238:A 2272-2283:A	LDSARSLTGQLMQFGSTL(abcdefgabcdefgabcd) LASIYYRFYDLTAARC(gabcdefgabcdefga) GESLLLNLAEME(abcdefgabcde)

6sz9	59-80:D 107-128:D 240-261:D	NVLNGIVIQQASDYHVYAQKLL(abcdefgabcdefgabcdefga) ERQRLVQLGDEFHKLELEQDNL(defgabcdefgabcdefgabcd) TNHLSQQAKSFRTQFYEVILRI(abcdefgabcdefgabcdefga)
6t46_ba1	133-144:G 149-163:G 173-187:G	AEFHYKIGVAYY(defgabcdefga) HLVSVNKVTKARDIY(abcdefgabcdefga) AIQCSLVVGINLYDM(efgabcdefgabcde)
6t46_ba1	170-184:G 190-207:G 212-227:G	NLEAIQCSLVVGINL(gabcdefgabcdefg) LDDADAYFRDALTEALDH(abcdefgabcdefgabcd) PITKIYHNLGLVHWQK(defgabcdefgabcde)
6tms	8-29:E 41-66:E 40-64:F	LRKLLEEAEKKLYKLEDKTRRS(defgabcdefgabcdefgabcd) AQSLQLIAESLMLIAESLLIIAISLL(defgabcdefgabcdefgabcdefga) KAQSLQLIAESLMLIAESLLIIAIS(efgabcdefgabcdefgabcdefga)
6vbu	135-146:4 151-164:4 170-181:4	WEICHNLGVCYI(defgabcdefga) FDKAQDQLHNALHL(abcdefgabcdefg) TYIMLGKIFLLK(abcdefgabcde)
6vbu	451-462:8 467-480:8 487-498:8	YEPHFNFATISD(defgabcdefga) LQRSYAAAKKSEAA(abcdefgabcdefg) TQHLIKQLEQHF(abcdefgabcde)
6w2q	68-82:A 91-105:A 113-127:A	PEAIIAAARALLKIA(defgabcdefgabcd) AKQAIEAASKAAQLA(abcdefgabcdefga) LVCEALALLIAAQVL(defgabcdefgabcd)
6wa0	6-26:A 5-26:B 9-26:C	LLVAFVAYYTALIALIFAILA(efgabcdefgabcdefgabcd) ALLVAFVAYYTALIALIFAILA(defgabcdefgabcdefgabcd) AFVAYYTALIALIFAILA(abcdefgabcdefgabcd)
6wc3	4-17:B 31-49:B 80-91:B	ATQLALLQDELLDM(abcdefgabcdefg) IIDKTLRFRELLGCYRLQV(defgabcdefgabcdefga) ALAQLLLWERFL(defgabcdefga)
6xf1	1432-1450:A 1460-1490:A 1512-1537:A	CIFKNNELLKNIQDVQSQI(defgabcdefgabcdefga) VPAVKHRKKSLIRLDKVLDEYEEEKRHLQEM(efgabcdefgabcdefgabcdefgabcdefg) TVVLWENTKALVTECLEQCGRVLELL(defgabcdefgabcdefgabcdefga)
6xns	192-206:B 221-235:B 251-265:B	LKAVETVVKVARALN(abcdefgabcdefga) AARVASEAARLAERV(defgabcdefgabcd) ARELQEKVLDILLDI(abcdefgabcdefga)

6xr1	3-24:A 32-54:A 62-80:A	LELALKALQILVNAAYVLAEIA(defgabcdefgabcdefgabcd) LLEKAARLAEEAARQAEEIARQA(gabcdefgabcdefgabcdefga) LALKALQILVNAAYVLAEI(defgabcdefgabcdefga)
6xt4_ba1	191-208:A 217-245:A 218-242:C	LLILILILLDLKEMLERL(abcdefgabcdefgabcd) IVKVLKVIVKAIEASVLNQAISAINQILL(abcdefgabcdefgabcdefgabcdefga) VKVLKVIVKAIEASVLNQAISAINQ(abcdefgabcdefgabcdefgabcd)
6zl1	16-27:C 50-61:C 96-107:C	IQKRIAAIQKRI(defgabcdefga) ITKQIAAIQLRI(defgabcdefga) IETQICKIEAAI(defgabcdefga)
7ag9	170-186:B 231-242:B 282-296:B	DDLINKNVEKCFEIQEL(efgabcdefgabcdefg) LSRKFLILKRNI(defgabcdefga) IMKNFRFMMNEIKDL(defgabcdefgabcd)
7ag9	309-330:B 344-362:B 375-389:B	FINLNNELEYIIEEVRLLLKKI(defgabcdefgabcdefgabcd) FNSQLAKKSKIITKTFNII(gabcdefgabcdefgabcd) IALKTNELAKVWVDL(defgabcdefgabcd)
7bjs	857-910:A 857-906:B 282-314:C	LENNLDQLTKVHKQLVRDNADLRCELPKLEKRLRCTMERVKALETALKEAKEGA(defgabcdefgabcdefgabcdefgabcdefgabcdefgabcdefgabcdefga) LENNLDQLTKVHKQLVRDNADLRCELPKLEKRLRCTMERVKALETALKEA(defgabcdefgabcdefgabcdefgabcdefgabcdefgabcdefgabcd) AESEVAALNRRIQLLEEDLERSEERLGSATAKL(defgabcdefgabcdefgabcdefgabcdefga)
7d3u	332-346:D 369-380:D 531-544:D	HHMIVKAALFLAIGA(defgabcdefgabcd) VAVAFFASAMSL(abcdefgabcde) AAPALALSVVTLAL(abcdefgabcdefg)
7dhg	187-198:C 203-216:C 224-235:C	VKALFRRAKAHE(defgabcdefga) KKECLEDVTAVCIL(abcdefgabcdefg) SMLLADKVLKLL(abcdefgabcde)
7drw	104-125:M 147-167:M 202-216:M	LALVKPEVWTLKEKCILVITWI(abcdefgabcdefgabcdefga) LERVNAVKTKVEAFQTTISKY(efgabcdefgabcdefgabcd) LRAMVLDLRAFYAEL(abcdefgabcdefga)
7kam	122-133:F 138-151:F 159-170:F	SGLYANRAQAHM(defgabcdefga) WPEGWVDAKCSVES(abcdefgabcdefg) GWWRGGKCLVEM(abcdefgabcde)
7n7p	12-65:A 83-94:A 12-61:B	CLRDWEDLQQDFQNIQETHRLYRLKLEELTKLQNNCTSSITRQKKRLQELALAL(defgabcdefgabcdefgabcdefgabcdefgabcdefgabcdefgabcdefga) LENQMKERQGLF(defgabcdefga) CLRDWEDLQQDFQNIQETHRLYRLKLEELTKLQNNCTSSITRQ

		KKRLQEL(defgabcdefgabcdefgabcdefgabcdefgabc defgabcdefgabcd)
7nj0	813-824:A 832-843:A 856-867:A	AGSSCHITQLLL(defgabcdefga) AQLHLEEAASSL(defgabcdefga) LSLTCDLLRSQL(defgabcdefga)
7o3v	122-158:B 168-213:B 169-205:C	MRRSIEERSRTAAATDKAVGLRAYEGAQQRLAQIEGL(cdefg abcdefgabcdefgabcdefgabcdefgabcd) QKAIEELQARIAGEQAAIQNETTKLQMIAQLRQAEQALISEQR RER(efgabcdefgabcdefgabcdefgabcdefgabcdefga bcdefga) KAIEELQARIAGEQAAIQNETTKLQMIAQLRQAEQAL(defga bcdefgabcdefgabcdefgabcdefgabcde)
7opb	2-13:D 21-32:D 42-53:D	VIEKLRKLEKQA(defgabcdefga) LVMLARMVLEYL(defgabcdefga) ADESADRIEEVL(defgabcdefga)
7qpg	20-38:W 59-77:W 95-113:W	LGTRISRLTRRVEEIKGEV(defgabcdefgabcdefga) LITQVDKLSEDIDLLKSRI(defgabcdefgabcdefga) LKQQLERDSVVLSLLKQLQ(defgabcdefgabcdefga)
7sil	620-631:A 655-668:A 838-849:A	AVLGIFLTAFVL(abcdefgabcde) LFSLLCCFSSSLFF(abcdefgabcdefg) VIAILAASFGLL(defgabcdefga)
7v1m	85-99:G 176-190:G 201-215:G	GEAFFFYGKSLLELA(defgabcdefgabcd) LELAWDMLDLAKIIF(abcdefgabcdefga) YAAQAHLKLGEVSVE(defgabcdefgabcd)
7vrb	7-18:A 63-80:A 89-107:A	QLHQLRAQIMAY(defgabcdefga) IQKMLDDNNHLIQCIMDS(defgabcdefgabcdefg) CSQYQQMLHTNLVYLATIA(defgabcdefgabcdefga)
7w5m	282-293:A 298-323:A 358-383:A	AELNFRICICLE(defgabcdefga) PKEAIPYCQKALLICKARMERLSNEI(abcdefgabcdefgab cdefgabcde) KEVEIGDLAGLAEDLEKKLEDLKQQA(abcdefgabcdefgab cdefgabcde)

Table S3. Sequences of *de novo* peptides and proteins in this study

Systematic name	Amino acid sequence	Mass (kDa)	ε
	gabcdef gabcdef gabcdef gabcdef		
apCCTri-A	Ac-G -LAALEK ELAA**T**EK ELAALEW ELAALE- G-NH$_2$	2979.58	5690 (ε$_{280}$)
apCCTri-B	Ac-G -LAALKE KLAALKE K**N**AALKY KLAALK- G-NH$_2$	2964.82	1280 (ε$_{275}$)
apCCTri-N	Ac-G KLAALEK KLAALEK K**T**AALEY KLAAL-- G-NH$_2$	2952.77	1280 (ε$_{275}$)
apCCTri-N’	Ac-G ELAALKE ELAALKE E**T**AALKY ELAAL-- G-NH$_2$	2955.62	1280 (ε$_{275}$)
apCCTri-A-c4CF	Ac-G -LAALEK ELAA**T**EK ELAALEQ ELAAL**Z**- G-NH$_2$	2964.58	32393 (ε$_{214}$)
apCCTri-A-n4CF	Ac-G **Z**LAALEK ELAA**T**EK ELAALEQ ELAALE- G-NH$_2$	3093.62	33394 (ε$_{214}$)
apCCTri-B-nMSE	Ac-G -**X**AALKE KLAALKE K**N**AALKQ KLAALK- G-NH$_2$	2995.72	28116 (ε$_{214}$)
apCCTri-N-nMSE	Ac-G K**X**AALEK KLAALEK K**T**AALEQ KLAAL-- G-NH$_2$	2982.63	28058 (ε$_{214}$)
X = Selenomethionine (MSE), Z = 4-cyanophenyalanine (4CF)			
sc-apCC3-ABN / CW1	MGSSHHHHHHSSGLVPRGSHM	12472.7	8250 (ε$_{280}$)
	MLAALKE KLAALKE K**N**AALKY KLAALK EKLGLTPE		
	LAALEK ELAA**T**EK ELAALEW ELAALE ADPNPDPA		
	KLAALEK KLAALEK K**T**AALEY KLAAL		
sc-apCC3-ABN / CW2	MGSSHHHHHHSSGLVPRGSHM	12503.6	8250 (ε$_{280}$)
	MLAALKE KLAALKE K**N**AALKY KLAALK KEKGETPE		
	LAALEK ELAA**T**EK ELAALEW ELAALE ADPNPDPA		
	KLAALEK KLAALEK K**T**AALEY KLAAL		
sc-apCC3-ABN’ / ACW	MGSSHHHHHHSSGLVPRGSHM	12486.6	8250 (ε$_{280}$)
	MLAALKE KLAALKE K**N**AALKY KLAALK KHKATPAE		
	LAALEK ELAA**T**EK ELAALEW ELAALE KKEPLTP		
	ELAALKE ELAALKE E**T**AALKY ELAAL		
	MGSSHHHHHHSSGLVPRGSHM		

sc-apCC3-CW-flex	MLAALKE KLAALKE KNAALKY KLAALK GSGSGS<br>LAALEK ELAATEK ELAALEW ELAALE GSGSGSGS<br>KLAALEK KLAALEK KTAALEY KLAAL	11964.9	8250 (ε_{280})
sc-apCC3-ACW-flex	MGSSHHHHHHSSGLVPRGSHM<br>MLAALKE KLAALKE KNAALKY KLAALK GSGSGS<br>LAALEK ELAATEK ELAALEW ELAALE GSGSGSGS<br>ELAALKE ELAALKE ETAALKY ELAAL	11967.7	8250 (ε_{280})
sc-apCC3-CW-mismatch	MGSSHHHHHHSSGLVPRGSHM<br>MLAALKE KLAALKE KNAALKY KLAALK AEKATPEE<br>LAALEK ELAATEK ELAALEW ELAALE KKEPLTP<br>KLAALEK KLAALEK KTAALEY KLAAL	12483.8	8250 (ε_{280})
sc-apCC3-ACW-mismatch	MGSSHHHHHHSSGLVPRGSHM<br>MLAALKE KLAALKE KNAALKY KLAALK EKLGLTPE<br>LAALEK ELAATEK ELAALEW ELAALE ADPNPDPA<br>ELAALKE ELAALKE ETAALKY ELAAL	12506.5	8250 (ε_{280})
sc-apCC3-CW-0NTT	MGSSHHHHHHSSGLVPRGSHM<br>MLAALKE KLAALKE KLAALKY KLAALK EKLGLTPE<br>LAALEK ELAALEK ELAALEW ELAALE ADPNPDPA<br>ELAALKE ELAALKE ELAALKY ELAAL	13087	8250 (ε_{280})
sc-apCC3-CW-2NTT	MGSSHHHHHHSSGLVPRGSHM<br>MLAALKE K**N**AALKE K**N**AALKY KLAALK EKLGLTPE<br>LAALEK ELAA**T**EK ELAA**T**EW ELAALE ADPNPDPA<br>KLAALEK K**T**AALEK K**T**AALEY KLAAL	12449.5	8250 (ε_{280})
sc-apCC3-CW-3NTT	MGSSHHHHHHSSGLVPRGSHM<br>M**N**AALKE K**N**AALKE K**N**AALKY KLAALK EKLGLTPE<br>LAALEK ELAA**T**EK ELAA**T**EW ELAA**T**E ADPNPDPA<br>E**T**AALEK E**T**AALEK E**T**AALKY ELAAL	12426.3	8250 (ε_{280})
sc-apCC3-CW-4NTT	MGSSHHHHHHSSGLVPRGSHM<br>M**N**AALKE K**N**AALKE K**N**AALKY K**N**AALK EKLGLTPE<br>LAA**T**EK ELAA**T**EK ELAA**T**EW ELAA**T**E ADPNPDPA<br>E**T**AALEK E**T**AALEK E**T**AALKY E**T**AAL	12403.2	8250 (ε_{280})

Systematic name	synthesis / host organism	gene synthesis	Insertion site	Vector	Insert length	Construct length
sc-apCC3-CW1	Escherichia coli	clonal	NdeI_XhoI	pET-28a(+)	308	5600
sc-apCC3-CW2	Escherichia coli	clonal	NdeI_XhoI	pET-28a(+)	308	5600
sc-apCC3-ACW	Escherichia coli	clonal	NdeI_XhoI	pET-28a(+)	309	5601
sc-apCC3-CW-flex	Escherichia coli	clonal	NdeI_XhoI	pET-28a(+)	309	5601
sc-apCC3-ACW-flex	Escherichia coli	clonal	NdeI_XhoI	pET-28a(+)	309	5601
sc-apCC3-CW-mismatch	Escherichia coli	clonal	NdeI_XhoI	pET-28a(+)	309	5601
sc-apCC3-ACW-mismatch	Escherichia coli	clonal	NdeI_XhoI	pET-28a(+)	309	5601
sc-apCC3-CW-0NTT	Escherichia coli	clonal	NdeI_XhoI	pET-28a(+)	312	5604
sc-apCC3-CW-2NTT	Escherichia coli	clonal	NdeI_XhoI	pET-28a(+)	312	5604
sc-apCC3-CW-3NTT	Escherichia coli	clonal	NdeI_XhoI	pET-28a(+)	312	5604
sc-apCC3-CW-4NTT	Escherichia coli	clonal	NdeI_XhoI	pET-28a(+)	312	5604

Systematic name	DNA sequence
sc-apCC3-CW-1	ATGCTTGCAGCATTAAAGGAGAAACTCGCAGCGCTGAAAGAGAAGA<br>ATGCGGCTCTGAAATACAAACTCGCCGCCCTGAAAGAGAAGTTGGG<br>GCTGACTCCTGAGTTGGCAGCCCTTGAGAAAGAGTTAGCGGCGAC<br>TGAGAAGGAACTGGCGGCCCTGGAATGGGAGTTGGCCGCTTTAGA<br>GGCAGACCCAAATCCTGATCCAGCGAAGTTAGCTGCCCTTGAGAAG<br>AAGTTGGCAGCACTGGAGAAGAAGACGGCAGCCCTGGAGTACAAG<br>CTTGCAGCGCTCTGA
sc-apCC3-CW-2	ATGCTCGCAGCACTCAAGGAGAAACTTGCAGCCTTAAAGGAAAAGA<br>ACGCGGCCCTCAAGTACAAGTTAGCAGCTTTGAAGAAAGAAAAGGG<br>TGAGACACCTGAACTGGCTGCGCTGGAGAAGGAATTAGCCGCGAC<br>CGAGAAAGAGTTGGCAGCACTGGAATGGGAACTCGCAGCTTTAGA<br>GGCCGATCCTAACCCAGACCCTGCAAAGCTCGCGGCCTTAGAAAA<br>GAAGCTCGCTGCCTTAGAAAAGAAGACAGCCGCGCTTGAGTACAAG<br>CTTGCGGCGCTGTGA
sc-apCC3-ACW	ATGCTGGCAGCTCTTAAAGAAAAACTGGCAGCACTCAAAGAAAAGA<br>ACGCAGCTCTGAAGTACAAACTGGCTGCTCTTAAAAAACATAAAGC<br>CACGCCAGCGGAACTGGCAGCATTGGAAAAAGAGCTGGCCGCCAC<br>AGAAAAGGAGCTGGCGGCATTAGAGTGGGAATTAGCGGCCCTTGA<br>AAAAAAAGAACCGCTTACTCCCGAATTGGCAGCCTTGAAAGAAGAA<br>TTGGCAGCCTTAAAAGAAGAGACAGCGGCCCTGAAGTATGAACTTG<br>CGGCACTGTGA
sc-apCC3-CW-flex	ATGCTTGCGGCACTCAAAGAGAAATTAGCAGCATTAAAGGAAAAGA<br>ACGCCGCTCTGAAGTACAAATTAGCTGCCCTGAAAGGCTCTGGTAG<br>CGGTAGTGAGCTGGCTGCACTCGAAAAAGAACTGGCGGCGACGGA<br>AAAGGAACTGGCGGCTCTCGAATGGGAACTGGCTGCACTTGAAGG<br>GTCAGGCTCGGGTTCCGGAAGCAAGCTGGCCGCTCTGGAAAAGAA<br>ACTGGCGGCGCTGGAGAAGAAAACCGCTGCGTTAGAATATAAACTC<br>GCCGCGCTGTGA

sc-apCC3-ACW-flex	ATGCTGGCTGCCCTGAAGGAGAAGCTGGCGGCTCTGAAAGAAAAG<br>AACGCCGCGCTTAAATACAAGCTCGCTGCGTTAAAAGGTAGCGGAT<br>CCGGTAGTGAACTCGCCGCTCTTGAAAAGGAACTTGCAGCGACGG<br>AAAAAGAATTGGCGGCATTAGAGTGGGAACTCGCCGCACTGGAAG<br>GCAGCGGCTCTGGTAGCGGCTCCGAACTGGCGGCACTTAAAGAAG<br>AATTAGCTGCACTTAAGGAAGAAACAGCCGCGTTGAAGTATGAACT<br>GGCGGCTCTTTAA
sc-apCC3-CW-mismatch	ATGTTGGCCGCGTTAAAAGAAAAACTTGCTGCGCTGAAAGAAAAAA<br>ACGCGGCTTTAAAATATAAGTTGGCGGCATTAAAAAAGCATAAGGC<br>GACTCCTGCCGAATTAGCAGCCCTTGAAAAAGAATTAGCGGCCACC<br>GAGAAAGAACTGGCGGCACTGGAATGGGAATTGGCGGCGCTGGAG<br>AAGAAGGAGCCTCTGACACCGAAATTAGCGGCATTGGAGAAAAAAT<br>TGGCCGCGCTCGAAAAAAAAACTGCGGCGTTAGAATATAAACTGGC<br>CGCGCTTTAG
sc-apCC3-ACW-mismatch	ATGCTGGCCGCCCTCAAAGAGAAACTTGCGGCACTCAAAGAGAAGA<br>ACGCGGCGCTGAAATATAAATTAGCCGCACTGAAAAAAGAAAAAGG<br>GGAGACTCCTGAGCTGGCGGCACTGGAAAAAGAACTGGCGGCCAC<br>GGAGAAAGAACTGGCGGCGTTAGAGTGGGAGCTGGCCGCCCTGG<br>AAGCCGATCCAAATCCAGATCCCGCTGAGCTGGCAGCCCTTAAAGA<br>GGAATTGGCTGCGTTAAAGGAGGAAACGGCGGCGCTGAAATATGA<br>ACTGGCAGCTTTGTAA
sc-apCC3-CW-0NTT	ATGCTCGCGGCGCTGAAAGAGAAGCTGGCCGCGCTCAAAGAGAAG<br>CTGGCCGCACTTAAATACAAGCTGGCAGCCCTGAAAGAGAAATTAG<br>GCTTAACACCAGAGCTCGCTGCACTCGAAAAGGAACTGGCCGCTCT<br>TGAAAAAGAACTGGCAGCGCTCGAATGGGAGTTAGCTGCACTGGAA<br>GCTGATCCGAACCCAGATCCGGCGAAGCTGGCGGCCCTCGAAAAA<br>AAGCTTGCGGCGTTAGAAAAGAAGCTGGCCGCGCTGGAATATAAAC<br>TCGCAGCTCTTTAA

sc-apCC3-CW-2NTT	ATGCTTGCAGCGCTGAAGGAAAAAAATGCGGCACTTAAAGAAAAAAATGCAGCTCTGAAATATAAACTCGCCGCACTGAAGGAAAAACTGGGTTTGACTCCGGAACTGGCGGCTCTGGAGAAGGAATTGGCAGCCACAGAGAAGGAGCTGGCCGCTACAGAGTGGGAACTTGCTGCACTGGAGGCAGATCCGAATCCTGATCCTGCCAAATTAGCCGCACTTGAGAAGAAAACGGCGGCTCTTGAAAAAAAAACGGCAGCCCTTGAGTACAAACTGGCAGCTCTTTGA
sc-apCC3-CW-3NTT	ATGAATGCGGCCTTGAAAGAGAAGAATGCCGCACTGAAGGAAAAAAACGCGGCACTGAAGTACAAACTTGCCGCCTTAAAGGAGAAGCTGGGTTTGACCCCTGAACTCGCAGCTCTTGAGAAAGAACTGGCAGCAACCGAAAAAGAATTAGCCGCGACCGAGTGGGAGCTGGCGGCAACAGAGGCGGACCCTAACCCCGATCCAGCCAAAACCGCGGCTTTAGAAAAAAAAACGGCTGCACTGGAAAAAAAAACTGCCGCGCTGGAATATAAACTGGCCGCCCTGTAA
sc-apCC3-CW-4NTT	ATGAATGCAGCACTTAAAGAAAAAAACGCCGCATTAAAGGAAAAAAATGCCGCCTTGAAATATAAAAACGCTGCGCTGAAAGAAAAACTCGGTCTTACACCGGAATTAGCCGCCACAGAGAAAGAATTAGCCGCAACTGAGAAAGAACTGGCCGCAACAGAATGGGAATTAGCGGCTACCGAAGCCGACCCCAATCCGGATCCGGCTAAGACAGCAGCTCTCGAAAAGAAAACGGCGGCTCTGGAAAAGAAAACCGCAGCCCTTGAATATAAAACTGCGGCCTTGTAA

Table S4. Evaluated Alphafold2 metrics for peptide assemblies, the unique 3-helix off-target combinations and proteins.

Model	average pLDDT	pTM	max pAE	Average pAE	*i*pTM
peptide mix AAA	96.55	0.88	16.42	2.21	0.87
peptide mix AAB	95.95	0.85	21.28	2.54	0.83
peptide mix NNN	95.50	0.85	22.13	2.54	0.83
peptide mix ABB	95.20	0.85	22.33	2.81	0.83
peptide mix ABN	95.18	0.85	23.39	2.69	0.83
peptide mix AAN	95.09	0.85	20.36	2.71	0.83
peptide mix ANN	94.81	0.84	23.64	2.71	0.82
peptide mix BBB	94.71	0.85	23.94	2.84	0.83
peptide mix BNN	94.60	0.85	25.69	2.80	0.83
peptide mix BBN	94.14	0.85	22.42	2.90	0.83
sc-apCC3-ACW	85.20	0.65	30.98	10.06	
sc-apCC3-ACW-flex	84.37	0.67	31.30	10.33	
sc-apCC3-ACW-mismatch	83.57	0.65	31.31	10.17	
sc-apCC3-CW1	85.31	0.65	31.25	9.35	
sc-apCC3-CW2	85.65	0.65	31.30	9.36	
sc-apCC3-CW-flex	82.48	0.65	31.31	10.67	
sc-apCC3-CW-mismatch	79.39	0.63	31.30	9.97	
sc-apCC3-CW-0NTT	84.84	0.65	31.22	9.44	
sc-apCC3-CW-2NTT	85.51	0.65	31.25	9.30	
sc-apCC3-CW-3NTT	84.07	0.62	31.31	10.01	
sc-apCC3-CW-4NTT	83.23	0.68	31.06	8.78	

Table S5. MASTER hits (match for helical regions) for each of the designed proteins in this study

Systematic name	MASTER hit PDB id	Hit residues	RMSD (Å)
sc-apCC3-CW	Loop1: 2fu2	29-35, 44-50	0.298
	Loop2: 3zn3	202-208, 217-223	0.350
sc-apCC3-ACW	Loop1: 3gae	628-635, 643-649	0.528
	Loop2: 2fu2	29-35, 43-49	0.495

Table S6. Summary of biophysical characterisation

systematic name	CD % helicity$_{222}$	T_M (°C)	SV weight (Observed / expected)	SE weight (Observed / expected)	Expected mass (Da)
Individual peptides					
peptide A	20.1	<10.0	n.d.	n.d.	2979.58
peptide B	42.7	<15.0	1.2	n.d.	2964.82
peptide N	78.7	79.0	2.5	2.3	2952.77
peptide N'	71.1	65.0	2.1	2.9	2955.62
Peptide assemblies (complex)					
apCCTri-ABN-CW	90.9	78.0	1.2	1.0	8897.17
apCCTri-ABN'-ACW	61.9	65.0	0.8	1.2	8900.02
Proteins					
sc-apCC3-CW1	78.5 ± 2.4	> 95.0	0.9	0.8	12472.7
sc-apCC3-CW2	70.8	> 95.0	1.0	n.d.	12503.6
sc-apCC3-CW-flex	68.7 ± 2.6	> 95.0	0.8	0.8	11964.9
sc-apCC3-CW-mismatch	57.1 ± 2.2	> 95.0	1.1	0.7	12483.8
sc-apCC3-ACW	82.2 ± 1.8	> 95.0	0.9	0.8	12486.6
sc-apCC3-ACW-flex	70.2 ± 2.0	> 95.0	0.8	0.8	11967.7
sc-apCC3-ACW-mismatch	53.5 ± 1.5	> 95.0	0.8	0.9	12506.5
sc-apCC3-CW-0NTT	75.1	> 95.0	2.2	0.8	13087.0
sc-apCC3-CW-2NTT	66.6	> 95.0	0.9	0.8	12449.5
sc-apCC3-CW-3NTT	62.6	86.5 ± 0.8	0.8	0.8	12426.3
sc-apCC3-CW-4NTT	36.3	25.3 ± 0.7	1.3	0.8	12403.2
Alternate states (peptides)					
peptide mix AAB	50.7	53.0	n.d.	n.d.	
peptide mix ABB	54.3	56.0	n.d.	n.d.	
peptide mix AAN	29.7	57.0	n.d.	n.d.	
peptide mix ANN	48.6	65.0	n.d.	n.d.	
peptide mix BNN	28.8	58.0	n.d.	n.d.	
peptide mix BBN	46.7	63.0	n.d.	n.d.	

Table S7. Crystallisation conditions used to obtain the X-ray structures discussed in this study

Systematic name	Molecular dimensions screen	Crystallisation conditions of the reservoir	Dilution sample / reservoir (/ seed) within the drop
sc-apCC3-CW-1	JCSG, E1	1.0 M sodium citrate tribasic dihydrate, 0.1 M sodium cacodylate, pH 6.5	2:1
sc-apCC3-CW-2	Proplex, G4	2.0 M ammonium sulfate, 0.1 M Tris, pH 8.0	2:1
sc-apCC3-CW-0NTT	Structure screen 1 and 2, G4	0.2 M Ammonium sulfate 0.1 M MES 30 % w/v PEG 5000 MME, pH 6.5	2:1
sc-apCC3-CW-2NTT	ProPlex, G12	1.0 M Sodium citrate tribasic dihydrate 0.1 M Sodium HEPES, pH 7.0	2:1
sc-apCC3-ACW	JCSG, H3	0.1 M BIS-Tris, 25 % *w/v* PEG 3350, pH 5.5	2:1:1

Table S8. Merging and refinement statistics for X-ray structures

	sc-apCC3-CW1	sc-apCC3-CW2	sc-apCCC3-ACW	sc-apCC3-CW-0NTT	sc-apCC3-CW-2NTT
PDB IDs	9rgv	9rgw	9rgx	9rgy	9rgz
Wavelength (Å)	0.9537 (13,000 eV)	0.9537 (13,000 eV)	0.6199 (20,001 eV)	0.6199 (20,001 eV)	0.9762 (12,701 eV)
Resolution range	36.82 - 2.1 (2.175 - 2.1)	50.18 - 2.15 (2.227 - 2.15)	36.97 - 2.2 (2.279 - 2.2)	32.22 - 1.9 (1.968 - 1.9)	40.12 - 2.05 (2.123 - 2.05)
Space group	C 1 2 1	P 41 21 2	P 21 21 21	C 1 2 1	I 1 2 1
Unit cell	95.832 37.866 77.432 90 122.206 90	63.358 63.358 246.633 90 90 90	37.9161 37.9161 147.87 90 90 90	107.409 37.1196 63.4334 90 112.617 90	72.7187 37.2862 83.821 90 106.83 90
Total reflections	26286 (2674)	56597 (5544)	144843 (14753)	125686 (12758)	89967 (8606)
Unique reflections	13624 (1380)	28306 (2772)	11473 (1126)	18511 (1829)	13719 (1345)
Multiplicity	1.9 (1.9)	2.0 (2.0)	12.6 (13.1)	6.8 (7.0)	6.6 (6.4)
Completeness (%)	97.09 (99.78)	99.38 (99.96)	99.98 (100.00)	99.92 (99.95)	99.59 (99.63)
Mean I/sigma(I)	10.89 (1.96)	32.56 (2.23)	8.87 (3.08)	10.34 (1.61)	13.56 (3.56)
Wilson B-factor (Å^2)	42.23	53.18	25.90	23.15	33.94
R-merge	0.0255 (0.306)	0.007184 (0.2151)	0.2102 (0.4033)	0.1007 (0.3515)	0.06717 (0.4526)
R-meas	0.03607 (0.4327)	0.01016 (0.3042)	0.2193 (0.4196)	0.1091 (0.3802)	0.07284 (0.4927)
R-pim	0.0255 (0.306)	0.007184 (0.2151)	0.06169 (0.1149)	0.04144 (0.144)	0.02786 (0.1923)
CC1/2	0.999 (0.845)	1 (0.886)	0.99 (0.885)	0.997 (0.938)	0.999 (0.941)
CC*	1 (0.957)	1 (0.969)	0.997 (0.969)	0.999 (0.984)	1 (0.985)
Reflections used in refinement	13600 (1378)	28302 (2771)	11473 (1126)	18502 (1828)	13708 (1344)
Reflections used for R-free	705 (61)	1403 (143)	621 (70)	923 (89)	660 (65)
R-work	0.2101 (0.2789)	0.2233 (0.2700)	0.2196 (0.2675)	0.1814 (0.2358)	0.2164 (0.2827)
R-free	0.2506 (0.3372)	0.2387 (0.3123)	0.2419 (0.2430)	0.2099 (0.2859)	0.2487 (0.3633)

CC(work)	0.971 (0.880)	0.965 (0.834)	0.876 (0.460)	0.960 (0.909)	0.966 (0.895)
CC(free)	0.977 (0.662)	0.970 (0.774)	0.790 (0.564)	0.946 (0.852)	0.981 (0.717)
Number of non-hydrogen atoms	1534	2264	1371	1789	1495
macromolecules	1384	2164	1289	1414	1353
ligands	0	11	0	0	0
solvent	150	89	82	375	142
Protein residues	196	300	192	193	195
RMS(bonds)	0.003	0.002	0.002	0.008	0.003
RMS(angles)	0.61	0.41	0.45	0.76	0.58
Ramachandran favoured (%)	100	100	97.31	100	100
Ramachandran allowed (%)	0	0	2.69	0	0
Ramachandran outliers (%)	0	0	0	0	0
Rotamer outliers (%)	0	1.12	4.65	0	0
Clashscore	3.58	0.91	4.75	5.14	4.84
Average B-factor	52.67	66.04	26.77	29.79	43.6
macromolecules	52.62	66.09	26.83	27.97	43.34
Ligand	-	96.6	-	-	-
solvent	53.19	61.1	25.5	36.68	46.09
Number of TLS groups	2	10	2	2	2

Table S9. Comparison of helicity predicted by DSSP analysis of AlphaFold2 models and experimentally measured helicity by CD spectroscopy. (*N* = No. of repeats)

protein name	DSSP calculated % helicity	experimentally measured fraction helicity
sc-apCC3-CW1	78.5	78.5 ± 2.4 (*N* = 3)
sc-apCC3-CW-flex	76.5	68.7 ± 2.6 (*N* = 3)
sc-apCC3-CW-mismatch	78.3	57.1 ± 2.2 (*N* = 3)
sc-apCC3-ACW	75.7	82.2 ± 1.8 (*N* = 3)
sc-apCC3-ACW-flex	73.9	70.2 ± 2.0 (*N* = 3)
sc-apCC3-ACW-mismatch	75.0	53.5 ± 1.5 (*N* = 3)
sc-apCC3-CW-0NTT	74.1	75.1
sc-apCC3-CW-2NTT	74.1	66.6
sc-apCC3-CW-3NTT	74.1	62.6
sc-apCC3-CW-4NTT	74.1	36.3

Table S10. Summary of thermodynamic analyses

[GdmHCl] (M)	% helicity	Fitted T_M (°C)
0.0	75.1	87.0
0.2	58.0	88.9
0.3	54.4	83.5
0.5	45.7	71.7
0.8	46.3	63.5
1.0	44.1	56.9
1.4	32.2	43.4
1.5	34.9	44.2
1.8	22.1	39.1

Globally fitted parameters:

ΔG (kcal mol^{-1})	6.16 ± 0.07
ΔH (kcal mol^{-1})	24.08 ± 0.53
ΔS (kcal mol^{-1} K^{-1})	0.06 ± < 0.01
ΔC_p (kcal mol^{-1} K^{-1})	0.19 ± 0.01
$\Delta\Delta C_p$ (kcal mol^{-1} K^{-1} M^{-1})	0.46 ± 0.01
m (kcal mol^{-1} M^{-1})	-3.22 ± 0.03

1.8 Supplementary figures

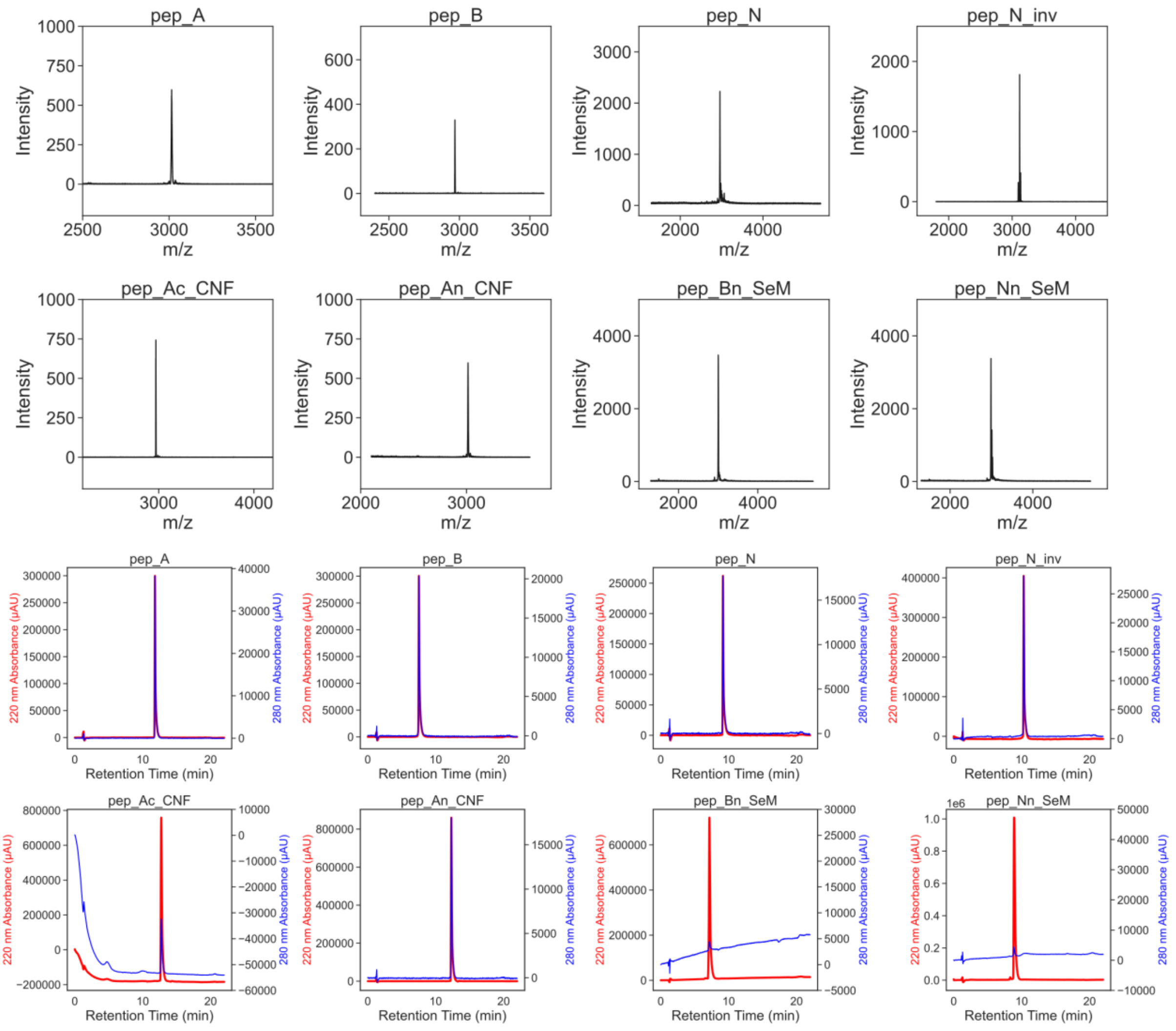

Fig. S1 MALDI-TOF spectra (top) and analytical HPLC traces (bottom) of *de novo* peptides (blue – 280 nm, red – 220 nm)

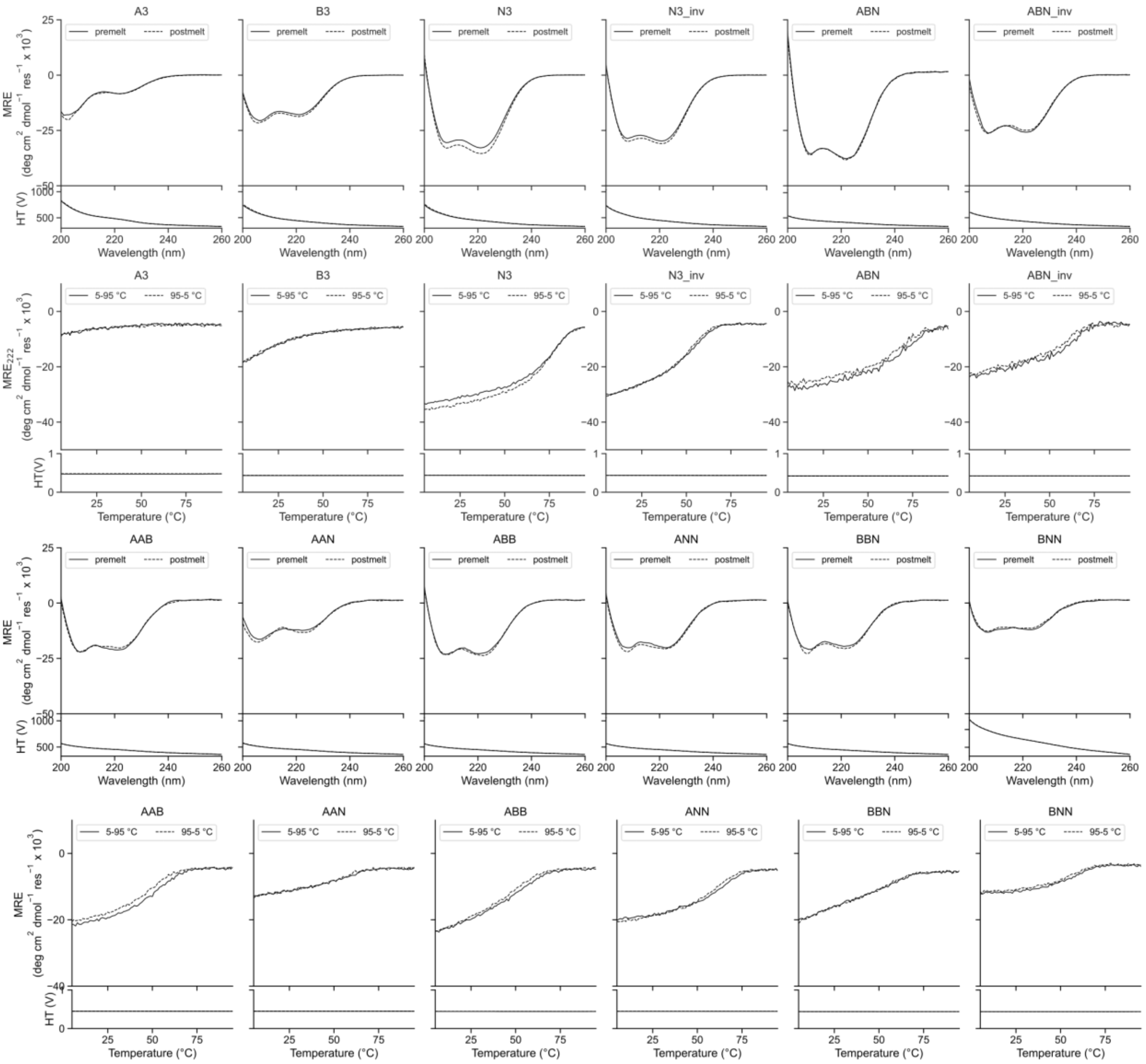

Fig. S2 CD spectra of the *de novo* peptides and the off-target mixtures

Sequences for individual peptides can be found in Supplementary Table 3. Summary of biophysical characterisation can be found in Supplementary Table 6. Conditions: 100 µM overall peptide assembly, PBS, pH 7.4, 5 to 95 °C. CD scans before the thermal ramps up (solid line) and after thermal ramps down (dashed line).

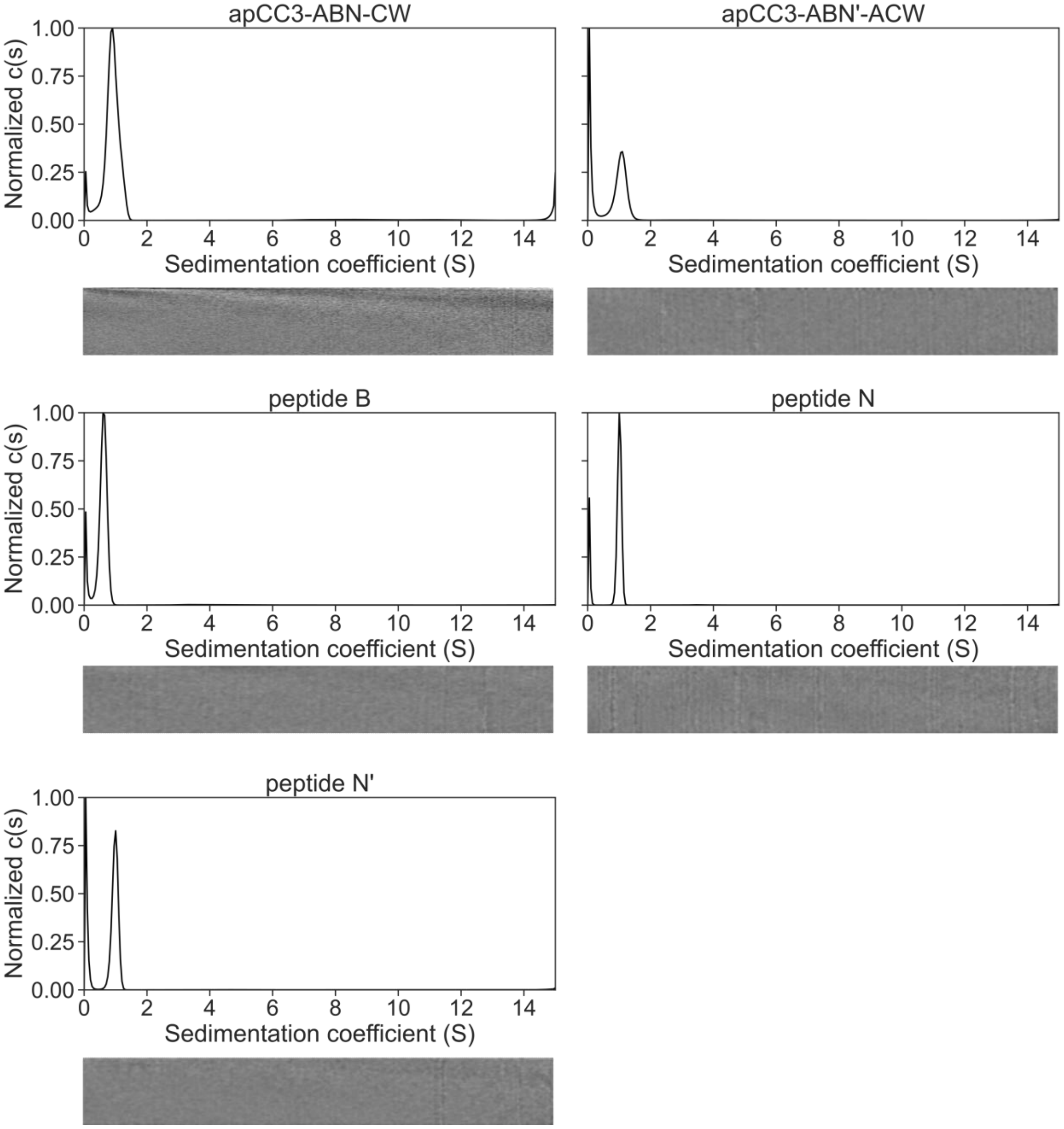

Fig. S3 AUC-SV traces of the *de novo* peptides

Sequences for individual peptides can be found in Supplementary Table 3. Summary of biophysical characterisation can be found in Supplementary Table 6. Residuals are shown as bitmaps below the fitted data. Conditions: 250 μM overall peptide assembly, PBS, pH 7.4, 20 °C, 60 krpm.

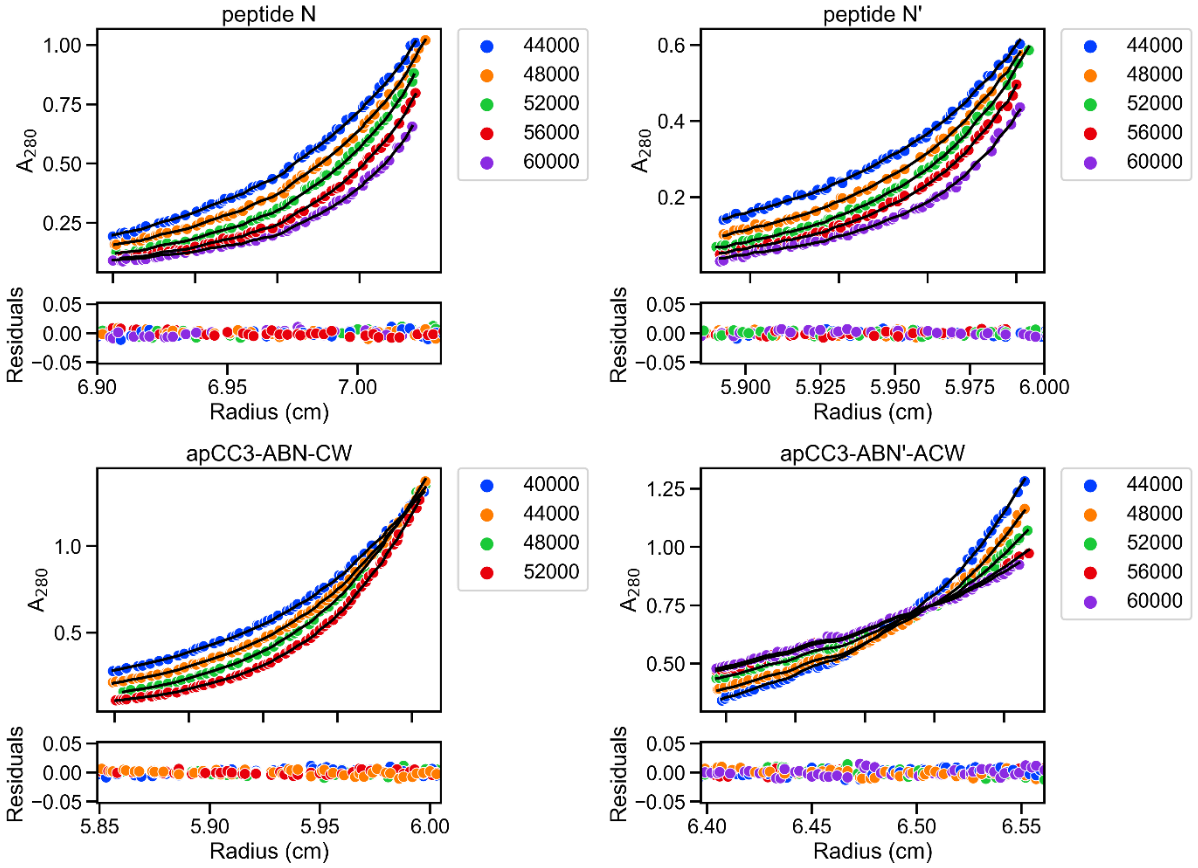

Fig. S4 AUC-SE traces of the *de novo* peptides

Sequences for individual peptides can be found in Supplementary Table 3. Summary of biophysical characterisation can be found in Supplementary Table 6. Residuals are shown as residual maps below the fitted data. Conditions: 250 μM overall peptide assembly, PBS, pH 7.4, 20 °C, 44-60 krpm with 4 krpm increment at 8 h intervals.

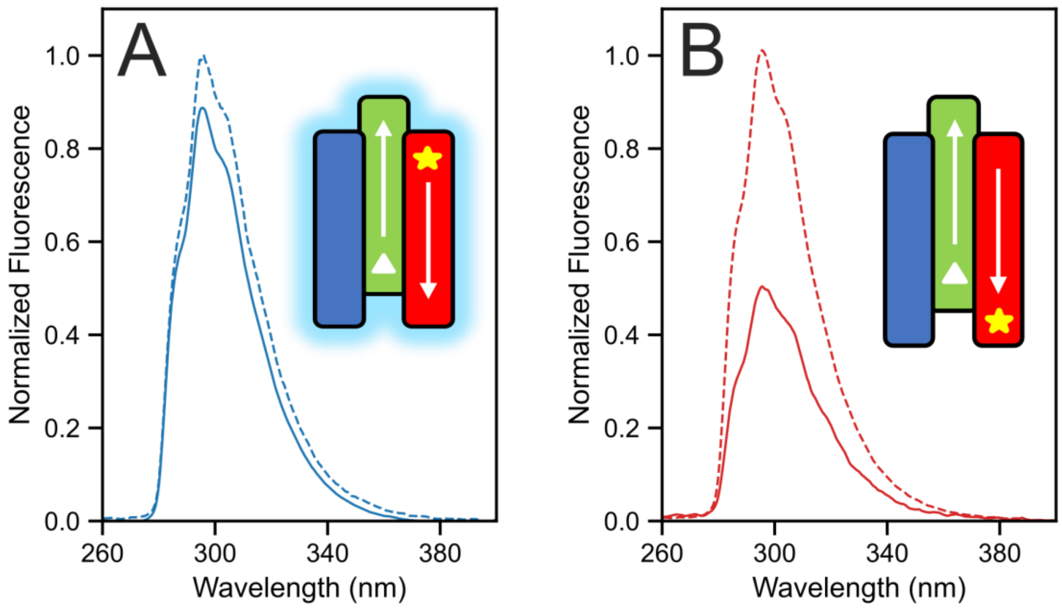

Fig. S5 Fluorescence-quenching assays for mixtures of apCC3-N-nMSE and apCC3-B plus apCC3-A-n4CF (A) or apCC3-A-c4CF (B). Dotted lines are for the fluorescently-labelled A peptides alone, and solid lines are for the 1:1:1 mixture. In the cartoons, arrows indicate the peptide direction from *N* to *C* termini; and 4CF is represented by the yellow star, and MSE by the white triangle. Conditions: 33 μM of each peptide in phosphate buffer (8.2 mM potassium phosphate dibasic, 1.8 mM potassium phosphate monobasic), pH 7.4.

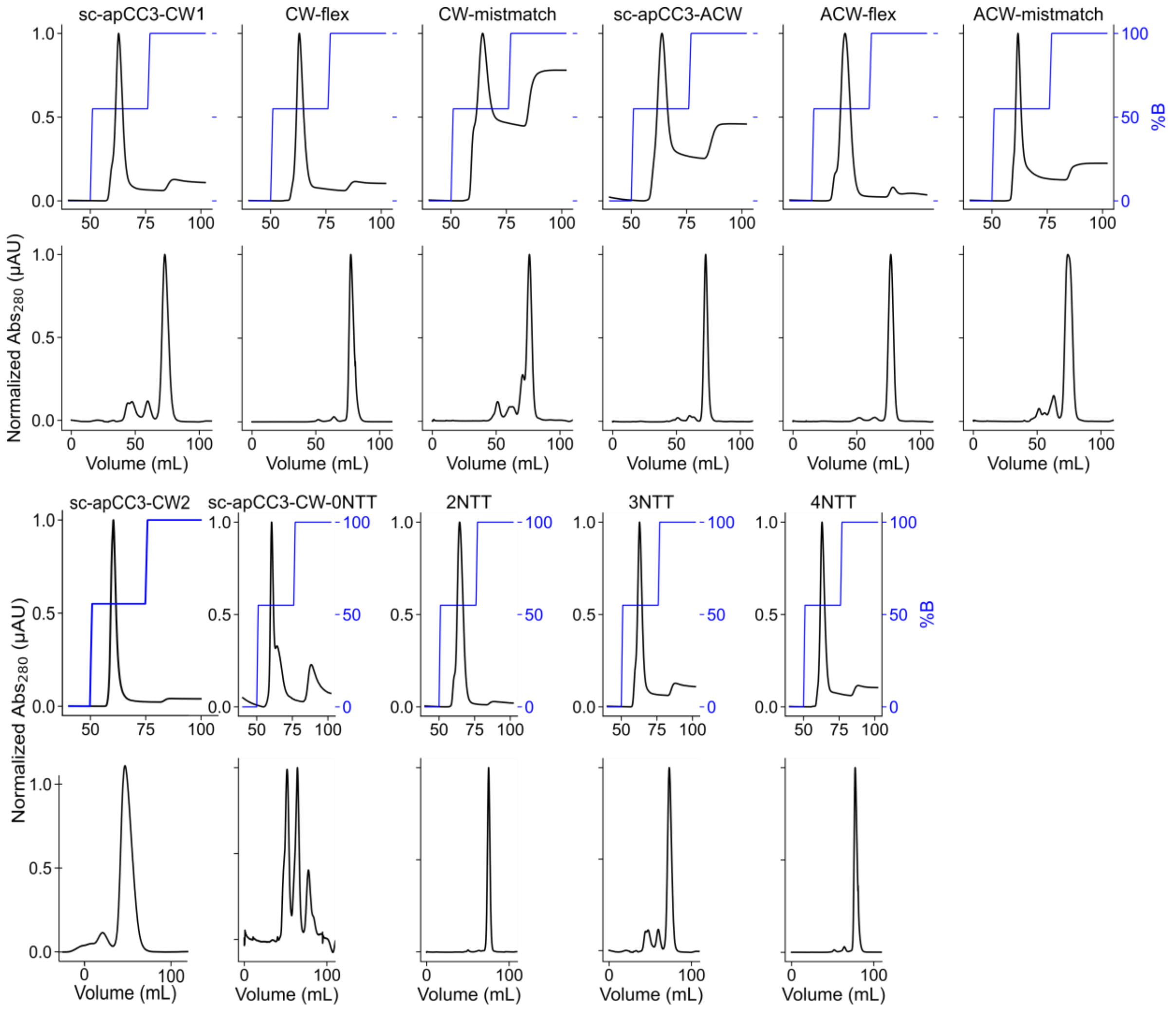

Fig. S6 FPLC purification of sc-apCC3 and its variants

Sequences for individual proteins can be found in Supplementary Table 3. Top: Ni-affinity chromatography. Fractions eluting at 55% B were pooled and concentrated then further purified by size-exclusion chromatography. Conditions: Buffer A: 50 mM Tris, pH 7.4, 500 mM NaCl, 30 mM imidazole, B: 300 mM imidazole. Bottom: Size-exclusion chromatography. Fractions eluting between 75-90 mL were pooled and concentrated for further biophysical analysis. Conditions: 50 mM sodium phosphate, pH 7.4, 150 mM NaCl.

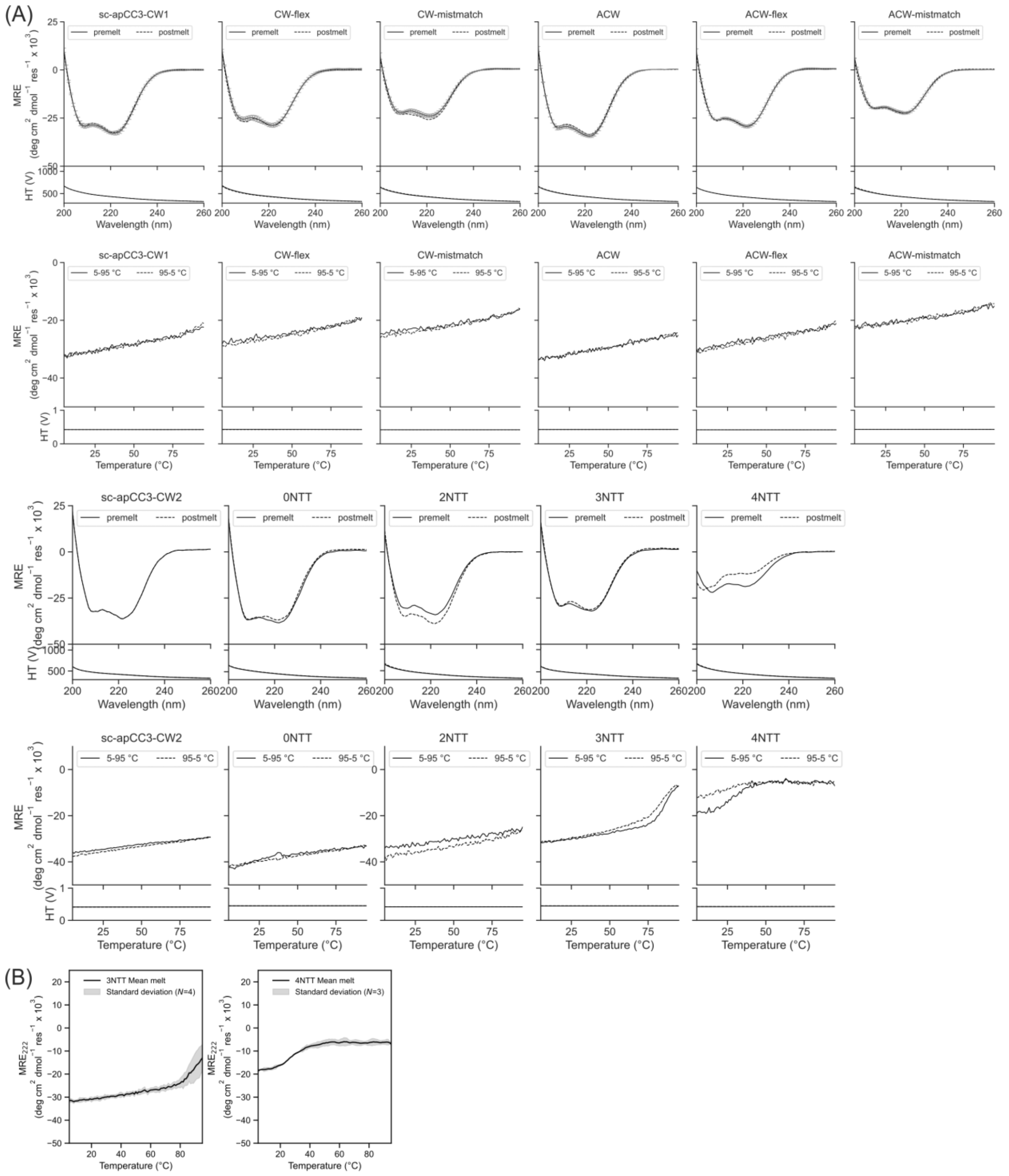

Fig. S7 CD spectra of the *de novo* proteins

Sequences for individual proteins can be found in Supplementary Table 3. Summary of biophysical characterisation can be found in Supplementary Table 6. (A) Conditions: 10 μM protein, 50 μM sodium phosphate, pH 7.4, 150 mM NaCl, top – 5 °C, error bars represent one standard deviation of the mean, *N*=3 for all loop variants; bottom – thermal response 5 to 95 °C. CD scans before the thermal ramps up (solid line) and after thermal ramps down (dashed line). (B) The filled regions represent one standard deviation of the mean; *N*=4 for 3NTT variant, *N*=3 for 4NTT variant**.**

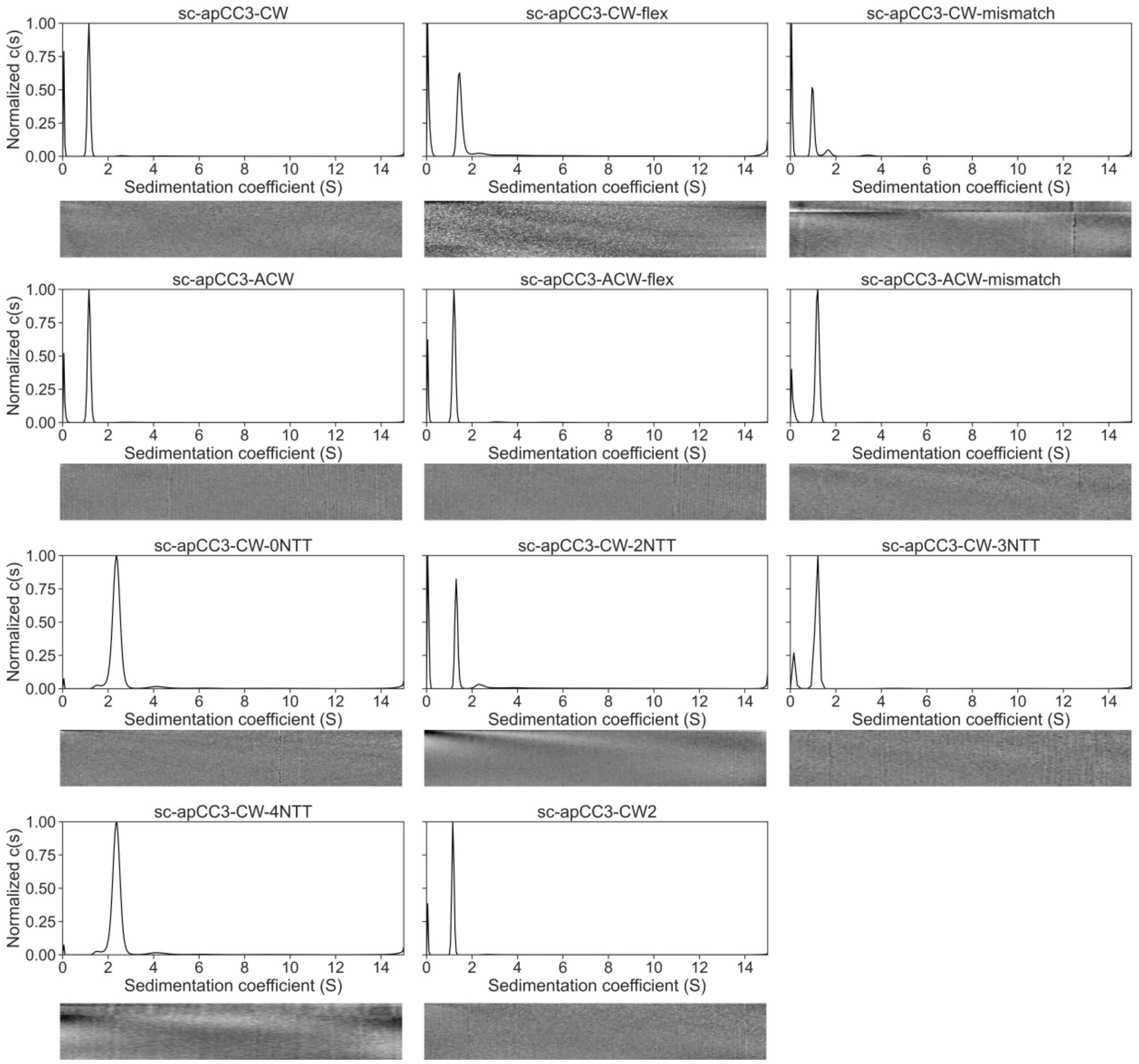

Fig. S8 AUC-SV traces of the *de novo* proteins

Sequences for individual proteins can be found in Supplementary Table 3. Summary of biophysical characterisation can be found in Supplementary Table 6. Residuals are shown as bitmaps below the fitted data. Conditions: 50 μM protein, 50 μM sodium phosphate, pH 7.4, 150 mM NaCl, 20 °C, 60 krpm.

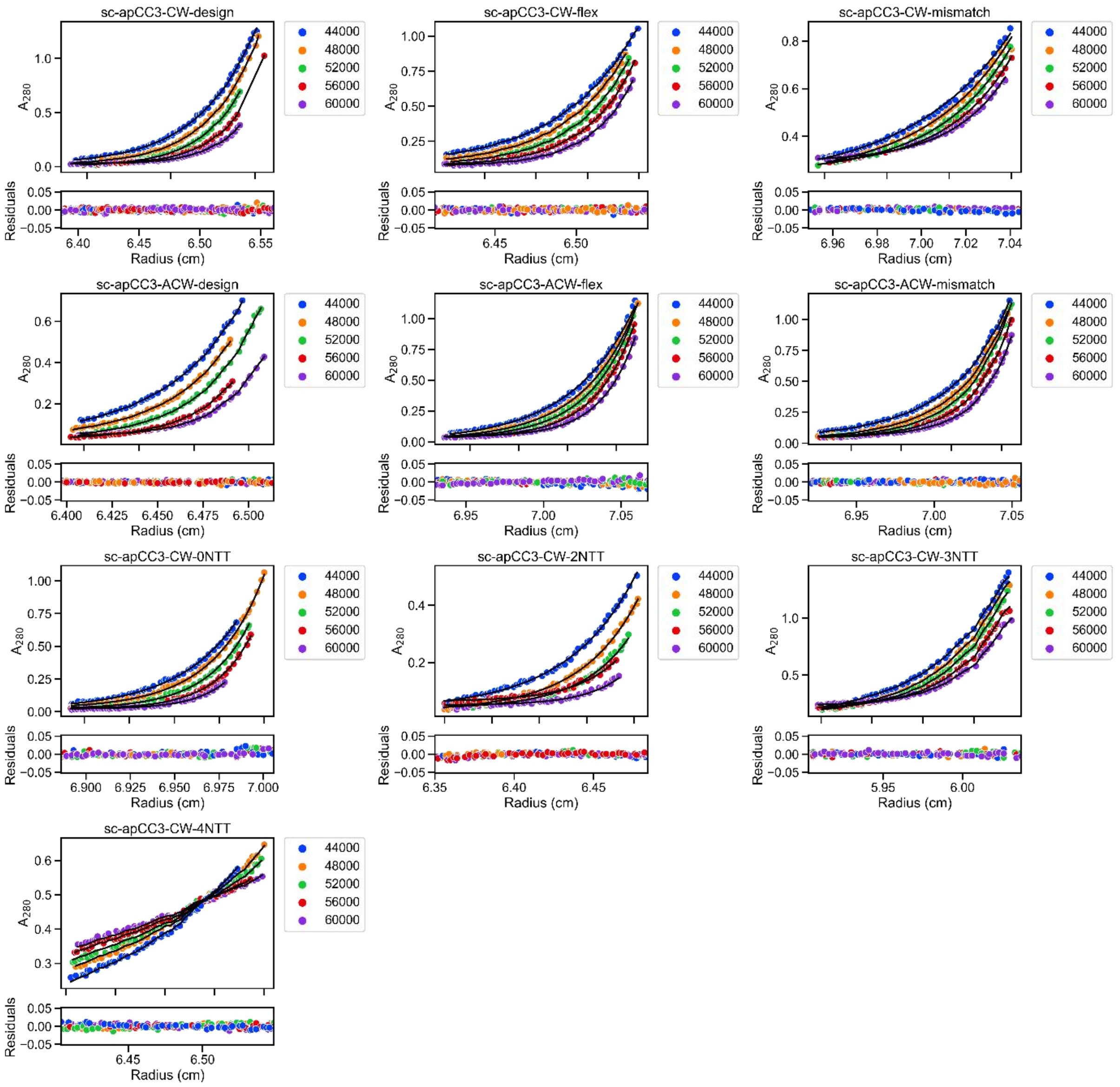

Fig. S9 AUC-SE traces of the *de novo* proteins

Sequences for individual proteins can be found in Supplementary Table 3. Summary of biophysical characterisation can be found in Supplementary Table 6. Residuals are shown as residual maps below the fitted data. Conditions: 50 μM protein, 50 μM sodium phosphate, pH 7.4, 150 mM NaCl, 20 °C, 44-60 krpm with 4 krpm increment at 8 h intervals.

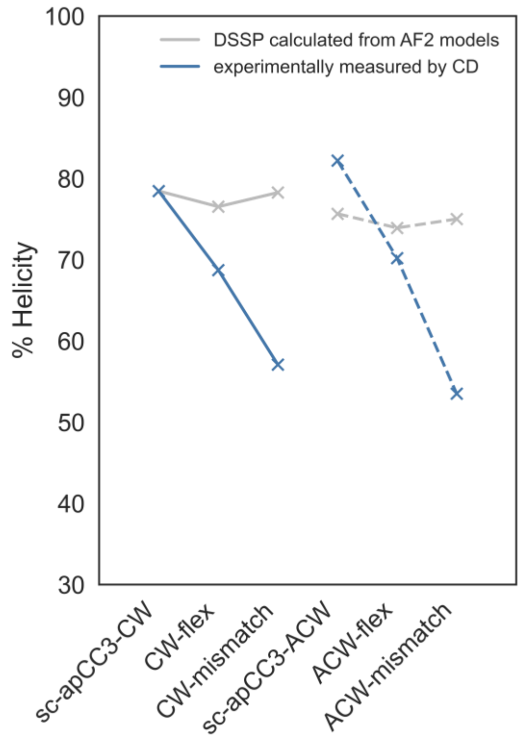

Fig. S10 Comparison of helicity predicted by DSSP analysis of AlphaFold2 models (grey) and experimentally measured helicity by CD spectroscopy (blue) across flexible and mismatched loop constructs. Key: variants with CW core sequences – solid lines, variants with ACW core sequences – dotted lines. Results for calculated helicities can be found in Supplementary Table S9.

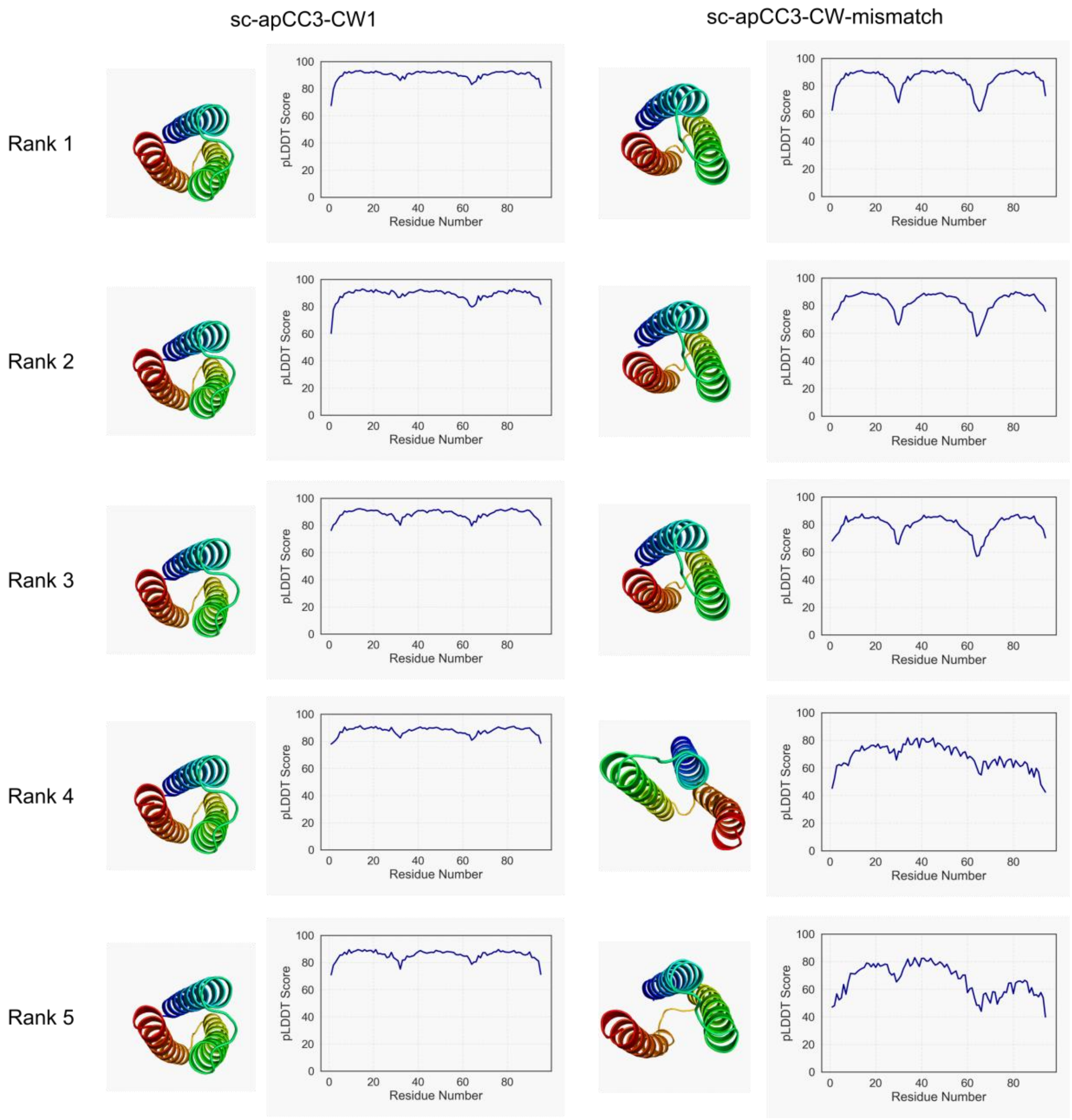

Fig. S11 AF2 predictions reveal potential access to alternative folds in mismatched constructs. Top-down views and pLDDT scores of AF2-predicted models for sc-apCC3-CW1 (left) all show consistent adoption of CW topology with high confidence score, whereas rank 4 and 5 predictions of sc-apCC3-CW-mismatch (right) show access to ACW topology and unfolding of the 3HB bundle, respectively. Mismatched sequences may challenge accurate global fold prediction.

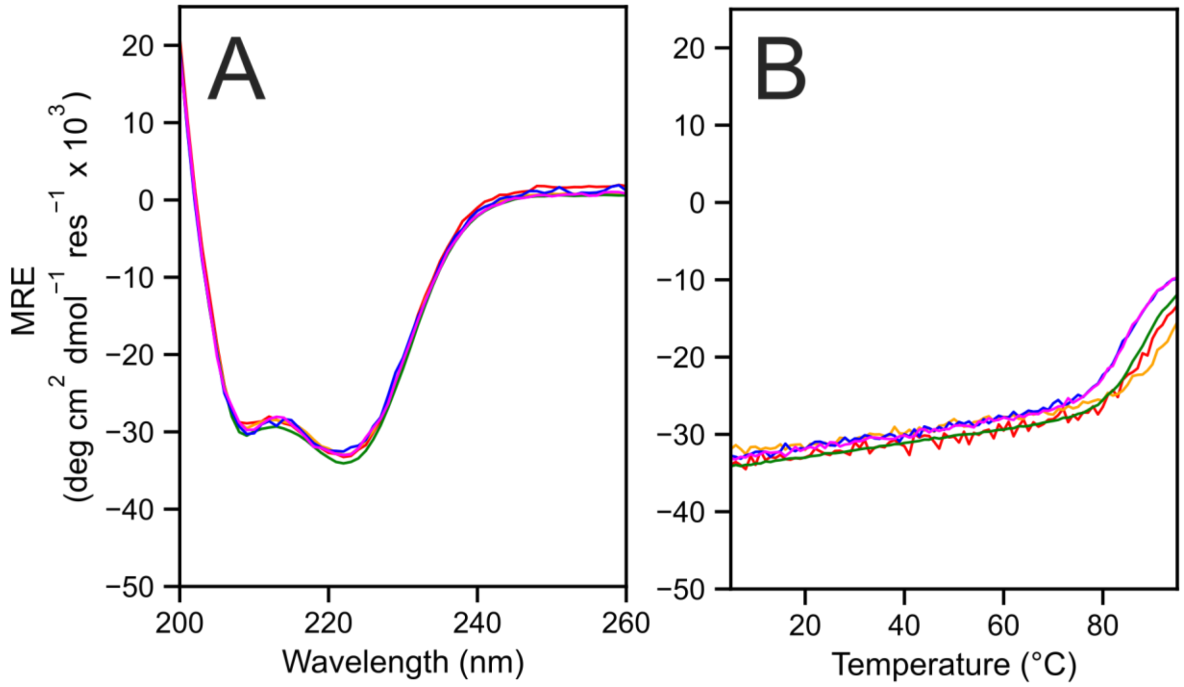

Fig. S12 CD measurements of sc-apCC3-3NTT at different concentrations, showing concentration-independent folding. Conditions: 50 μM sodium phosphate, pH 7.4, 150 mM NaCl, (A) CD spectra recorded at 5 °C, (B) thermal response of the CD signals at 222 nm (ramping from 5 to 95 °C at 1 °C min^{-1}). Key: 5 μM – red, 10 μM – yellow, 20 μM – green, 30 μM – blue, 45 μM – magenta.

Thermal denaturation modelling and thermodynamic analysis using van't Hoff equations

Each thermal denaturation curve was fitted to a two-state folding model, assuming there are only folded and unfolded states due to a sharp sigmoidal transition. The two-state folding thermodynamic parameters were extracted as described by Gellman[13] using Equations 1-9 and the following parameters.

T	absolute temperature
θ_f	ellipticity contribution from folded conformation
θ_u	ellipticity contribution from unfolded conformation
ΔH	enthalpy change at a given temperature
ΔS	entropy change at a given temperature
K	equilibrium constant
b_f	y-intercept of folded baseline
α	fraction folded conformation (0 ≤ α ≤ 1)
ΔG	Gibbs free energy change
ΔC_p	heat capacity change of folding
θ_T	measured ellipticity
M	melting temperature
T_r	reference temperature
m_f	slope of folded baseline
x	temperature
$\Delta\Delta C_p$	Denaturant concentration dependence of heat capacity of folding
R	universal gas constant
b_u	y-intercept of unfolded baseline

Equation 1 shows how the observed ellipticity θ_T is composed of a combination of signals from both the folded and unfolded states. The melting curve function models the temperature dependent transition between the two states by calculating the fraction folded conformation at a given temperature x using a sigmoidal function, Equations 2 and 3. The sigmoidal function describes how the folding transition occurs as the temperature changes, driven by enthalpy-like steepness term *h* and melting temperature M. The function uses a weighted sum of signals from both states, b_f and b_u, and m_f describes a linear slope at the folded state (Figure S13). The fraction of

folded conformation, α, is defined by rearranging the equilibrium constant of folding (Equation 4, Figure S14) in terms of the Gibbs free energy (Equations 5 and 6). This relationship forms the basis for the subsequent thermodynamic models.

$$\theta_T = \alpha\theta_f + (1-\alpha)\theta_u \qquad \text{Equation 1}$$

$$y(x) = f(x)\cdot\left(b_f - b_u + m_f x\right) + b_u \qquad \text{Equation 2}$$

$$\text{Where } f(x) = \frac{1}{1+\exp\left(-\frac{h\left(1-\frac{x}{M}\right)}{x}\right)} \qquad \text{Equation 3}$$

$$\alpha = \frac{1}{1+K} \qquad \text{Equation 4}$$

$$K = e^{-\frac{\Delta G}{RT}} \qquad \text{Equation 5}$$

$$\ln K = -\Delta G(T) \qquad \text{Equation 6}$$

$$\theta_f = b_f + m_f T + a[GdmHCl] \qquad \text{Equation 7}$$

$$\theta_u = b_u + m_u T + b[GdmHCl] \qquad \text{Equation 8}$$

To characterise the thermal stability of the system, we fitted all unfolding transitions assuming the following dependences. The ellipticities of folded and unfolded states linearly depend on temperature and concentration of denaturant concentration (Equations 7 and 8). The Gibbs free energy of the native state is determined by equation 9 (Fig S15, Table S10).[14,15]

$$\Delta G(T,[GdmHCl]) = \Delta H_0 - T\Delta S_0 + \left(\Delta C_{p,0} + \Delta\Delta C_{p,0}\cdot[GdmHCl]\right)\left(T - T_0 + T\ln\left(\frac{T_0}{T}\right)\right) + m[GdmHCl]$$

Equation 9

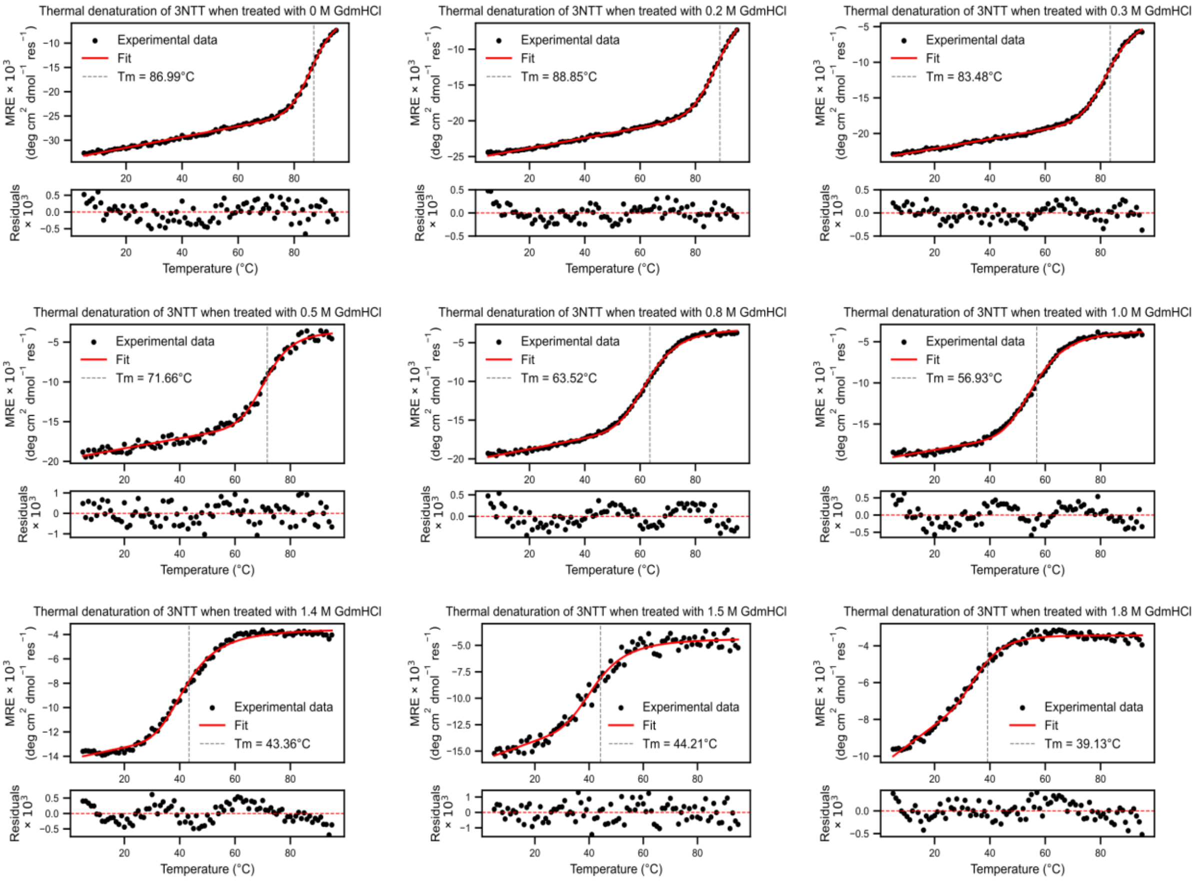

Fig. S13 Variable temperature CD signal of sc-apCC3-CW-3NTT when treated with GdmHCl concentrations of 0, 0.2, 0.3, 0.5, 0.8, 1.0, 1.4, 1.5, 1.8 M, monitored at 222 nm, 5 to 95 °C. Data was fitted to a two-state folding model (top) for melting temperature calculation and fit residual (bottom) using Equations 2 and 3. Results for individually fitted T_M can be found in Supplementary Table S10.

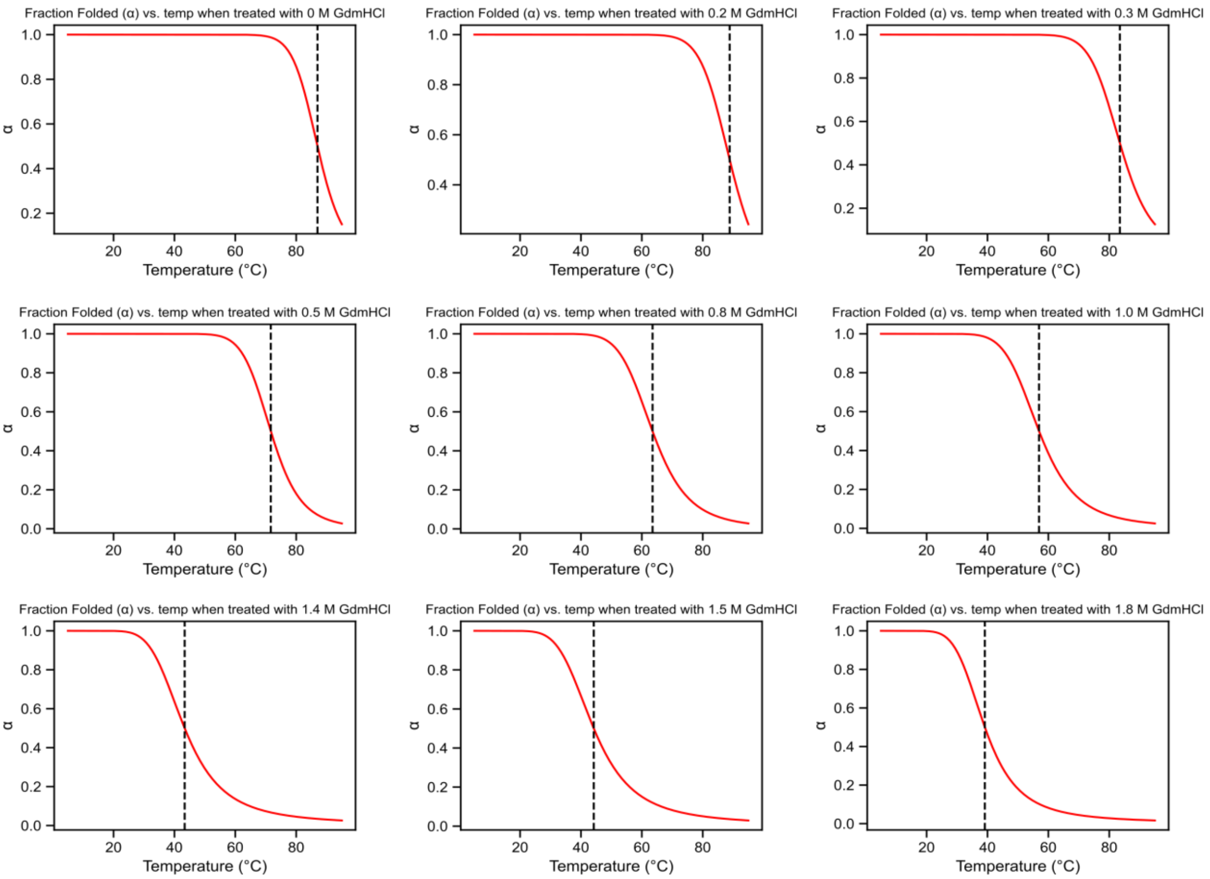

Fig. S14 Fraction folded (α) of sc-apCC3-3NTT unfolding as a function of temperature calculated using Equation 4. The dotted line represents the transition midpoint temperature when α = 0.5.

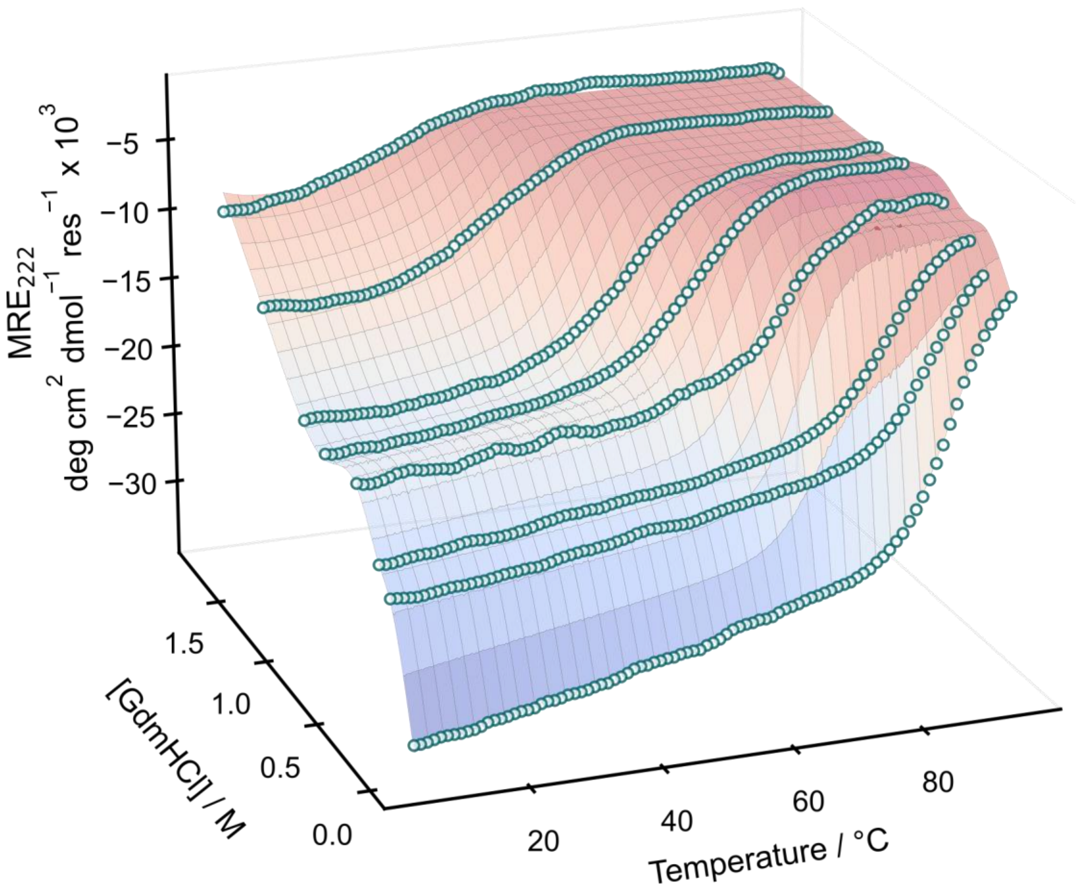

Fig. S15 MRE at 222 nm of sc-apCC3-3NTT unfolding as a function of temperature and GdmHCl concentration. Conditions: 10 μM protein, 50 μM sodium phosphate, 150 mM NaCl, pH 7.4, 5 to 95 °C; treated with GdmHCl concentrations of 0, 0.2, 0.3, 0.5, 0.8, 1.0, 1.4, 1.5, 1.8 M. Raw data are shown as blue scatters, and the globally-fitted thermodynamic parameters are represented by the surface. Errors are reported as fitting errors. Thermodynamic parameters were extracted using Equation 9 and results can be found in Supplementary Table S10.

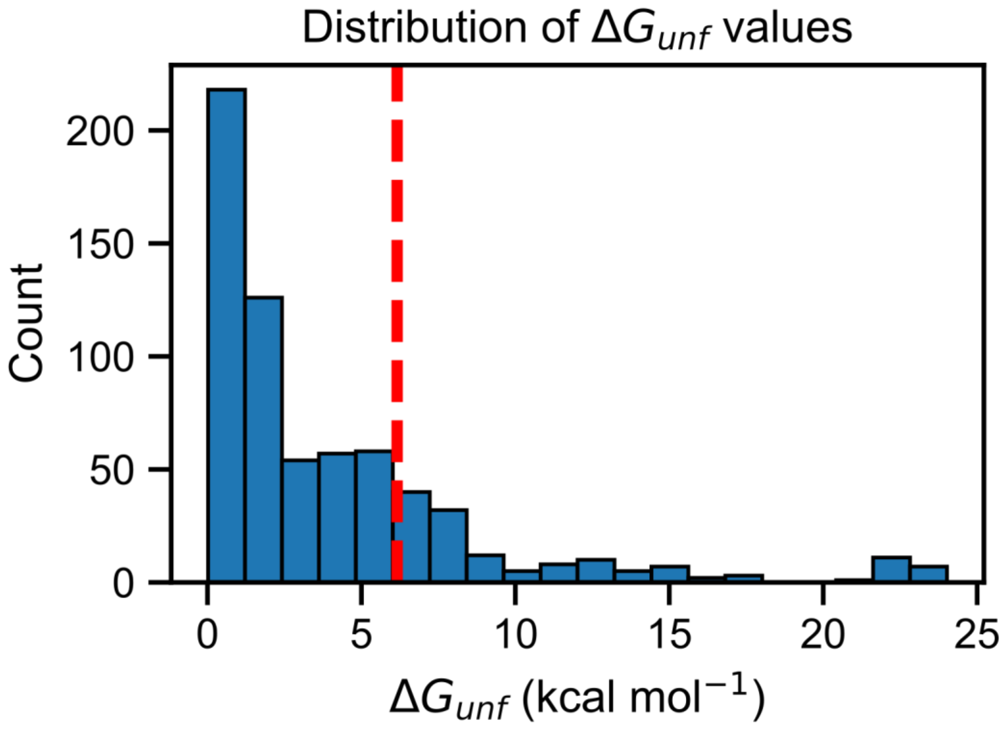

Fig. S16 Histogram of ΔG_{unf} in the ProTherm protein stability database[16] (search limited to helix, GdmHCl, yielded 656 examples). The estimated ΔG_{unf} of sc-apCC3-CW-3NTT (ΔG_{unf} = 6.16 ± 0.07 kcal mol^{-1}) reported at reference temperature of 25 °C is indicated by the red dashed line.

References

1. P. Kumar, R. Petrenas, W. M. Dawson, H. Schweke, E. D. Levy and D. N. Woolfson, *Protein Sci.*, 2023, **32**.
2. G. Kim, S. Lee, E. Levy Karin, H. Kim, Y. Moriwaki, S. Ovchinnikov, M. Steinegger and M. Mirdita, *Nature Protocols*, 2024, DOI: 10.1038/s41596-024-01060-5.
3. J. Jumper, R. Evans, A. Pritzel, T. Green, M. Figurnov, O. Ronneberger, K. Tunyasuvunakool, R. Bates, A. Žídek, A. Potapenko, A. Bridgland, C. Meyer, S. A. A. Kohl, A. J. Ballard, A. Cowie, B. Romera-Paredes, S. Nikolov, R. Jain, J. Adler, T. Back, S. Petersen, D. Reiman, E. Clancy, M. Zielinski, M. Steinegger, M. Pacholska, T. Berghammer, S. Bodenstein, D. Silver, O. Vinyals, A. W. Senior, K. Kavukcuoglu, P. Kohli and D. Hassabis, *Nature*, 2021, **596**, 583-589.
4. J. Zhou and G. Grigoryan, unpublished work.
5. J. Zhou and G. Grigoryan, *Protein Sci.*, 2015, **24**, 508-524.
6. J. Dauparas, I. Anishchenko, N. Bennett, H. Bai, R. J. Ragotte, L. F. Milles, B. I. M. Wicky, A. Courbet, R. J. d. Haas, N. Bethel, P. J. Y. Leung, T. F. Huddy, S. Pellock, D. Tischer, F. Chan, B. Koepnick, H. Nguyen, A. Kang, B. Sankaran, A. K. Bera, N. P. King and D. Baker, *Science*, 2022, **378**, 49-56.
7. W. Kabsch and C. Sander, *Biopolymers*, 1983, **22**, 2577-2637.
8. W. G. Touw, C. Baakman, J. Black, T. Beek, H., Tim A., E. Krieger, R. P. Joosten and G. Vriend, *Nucleic Acids Res.*, 2015, **43**, D364-D368.
9. B. J. H. Kuipers and H. Gruppen, *J. Agric. Food. Chem.*, 2007, **55**, 5445-5451.
10. M. D. Watson, I. Peran and D. P. Raleigh, *Biochemistry*, 2016, **55**, 3685-3691.
11. M. D. Winn, C. C. Ballard, K. D. Cowtan, E. J. Dodson, P. Emsley, P. R. Evans, R. M. Keegan, E. B. Krissinel, A. G. W. Leslie, A. McCoy, S. J. McNicholas, G. N. Murshudov, N. S. Pannu, E. A. Potterton, H. R. Powell, R. J. Read, A. Vagin and K. S. Wilson, *Acta Crystallogr. D*, 2011, **67**, 235-242.
12. P. V. Afonine, R. W. Grosse-Kunstleve, N. Echols, J. J. Headd, N. W. Moriarty, M. Mustyakimov, T. C. Terwilliger, A. Urzhumtsev, P. H. Zwart and P. D. Adams, *Acta Crystallogr. D*, 2012, **68**, 352-367.
13. D. F. Kreitler, D. E. Mortenson, K. T. Forest and S. H. Gellman, *J. Am. Chem. Soc.*, 2016, **138**.
14. N. A. Tavenor, Z. E. Reinert, G. A. Lengyel, B. D. Griffith and W. S. Horne, *Chem. Commun.*, 2016, **52**, 3789-3792.
15. Z. E. Reinert, G. A. Lengyel and W. S. Horne, *J. Am. Chem. Soc.*, 2013, **135**, 12528-12531.
16. R. Nikam, A. Kulandaisamy, K. Harini, D. Sharma and M. M. Gromiha, *Nucleic Acids Res.*, 2021, **49**, 420-424.
